# Network topology diversification of porous organic salts†

**DOI:** 10.1039/d4sc01218f

**Published:** 2024-05-01

**Authors:** Hiroi Sei, Kouki Oka, Yuta Hori, Yasuteru Shigeta, Norimitsu Tohnai

**Affiliations:** a Department of Applied Chemistry, Graduate School of Engineering, Osaka University 2-1 Yamadaoka, Suita Osaka 565-0871 Japan tohnai@chem.eng.osaka-u.ac.jp; b Institute of Multidisciplinary Research for Advanced Materials, Tohoku University 2-1-1 Katahira, Aoba-ku Sendai Miyagi 980-8577 Japan; c Center for Computational Sciences, University of Tsukuba 1-1-1 Tennodai Tsukuba Ibaraki 305-8577 Japan

## Abstract

Hydrogen-bonded organic frameworks (HOFs) are porous organic materials constructed *via* hydrogen bonds. HOFs have solubility in specific high-polar organic solvents. Therefore, HOFs can be returned to their components and can be reconstructed, which indicates their high recyclability. Network topologies, which are the frameworks of porous structures, control the pore sizes and shapes of HOFs. Therefore, they strongly affect the functions of porous materials. However, hydrogen bonds are usually weak interactions, and the design of the intended network topology in HOFs from their components has been challenging. Porous organic salts (POSs) are an important class of HOFs, are hierarchically constructed *via* strong charge-assisted hydrogen bonds between sulfonic acids and amines, and therefore are expected to have high designability of the porous structure. However, the network topology of POSs has been limited to only ***dia***-topology. Here, we combined tetrasulfonic acid with the adamantane core (4,4′,4′′,4′′′-(adamantane-1,3,5,7-tetrayl)tetrabenzenesulfonic acid; AdPS) and triphenylmethylamines with modified substituents in *para*-positions of benzene rings (TPMA-X, X = F, methyl (Me), Cl, Br, I). We changed the steric hindrance between the adamantane and substituents (X) in TPMA-X and obtained not only the common ***dia***-topology for POSs but also rare ***sod***-topology, and ***lon***- and ***uni***-topologies that are formed for the first time in HOFs. Changing template molecules under preparation helped in successfully isolating the porous structures of AdPS/TPMA-Me with ***dia***-, ***lon***-, and ***sod***-topologies which exhibited different gas adsorption properties. Therefore, for the first time, we demonstrated that the steric design of HOF components facilitated the formation, diversification, and control of the network topologies and functions of HOFs.

## Abstract 2

Strong charge-assisted hydrogen bonds and the conformation style of components achieved the proof-of-concept for the formation, diversification, and control of the network topologies and concomitant functions of hydrogen-bonded organic frameworks.

## Graphical abstract

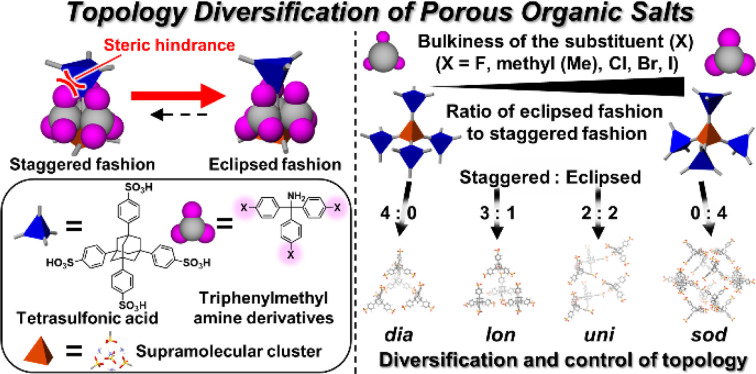

## Introduction

Organic porous materials are composed of organic molecules and can be facilely functionalized *via* the molecular design of their components.^1^ Moreover, they are metal-free and composed of abundant elements (C, N, O, S, *etc*.) on earth, and are therefore expected to have high environmental acceptability.^1*c*,2^ Representative organic porous materials, covalent organic frameworks (COFs) constructed *via* covalent bonds, and hydrogen-bonded organic frameworks (HOFs) constructed *via* hydrogen bonds, are well-known. They have been aggressively investigated based on the design of nanospaces toward various functions and applications such as selective adsorption and separation of gas molecules,^3^ stimulated response to specific ions and molecules,^4^ ion conduction,^5^ and catalytic reactions.^6^ Among them, HOFs can be prepared under mild conditions such as recrystallization, and continuous bond formation and dissociation allow them to eliminate energetically unfavorable defects and to construct thermodynamically stable ordered structures with high crystallinity.^7^ Single-crystal X-ray diffraction analysis enables us to reveal the detailed crystal structure, such as the framework of the porous structures (network topology), the interpenetration of the frameworks, and the bond lengths and angles, and therefore, to investigate a correlation between their porous structures and functions. In addition, HOFs are soluble in specific high-polar solvents, return to their components facilely, and then are reproduced *via* recrystallization, which exhibits their high recyclability.^8^

In general, network topologies control the pore sizes and shapes of the porous structures, and strongly affect their functions.^9^ Therefore, diversification and control of network topology are significantly important in the investigation of porous materials. The conventional studies of HOFs have aimed to form various network topologies by changing the combination between supramolecular synthons^1*a*,10^ which are the common patterns of hydrogen bonds in crystal structures, and tectons^10*b*,11^ which are the building blocks. However, the conventional HOFs are constructed by weak hydrogen bonds and form unintended bonds^12^ including the involvement of solvent molecules that are different from supramolecular synthons, which causes the construction of unexpected structures. Therefore, it is difficult to predict network topologies from supramolecular synthons and tectons.^8^ Some previous studies have reported that the change of the solvent condition in recrystallization enables HOFs to form porous structures with different network topologies.^13^ Furthermore, polymorphic and pseudopolymorphic porous structures of HOFs are often constructed even under the same solvent conditions in recrystallization.^14^ Therefore, isolating and selectively obtaining a porous structure with only a specific network topology has been difficult.

**Fig. 1:**
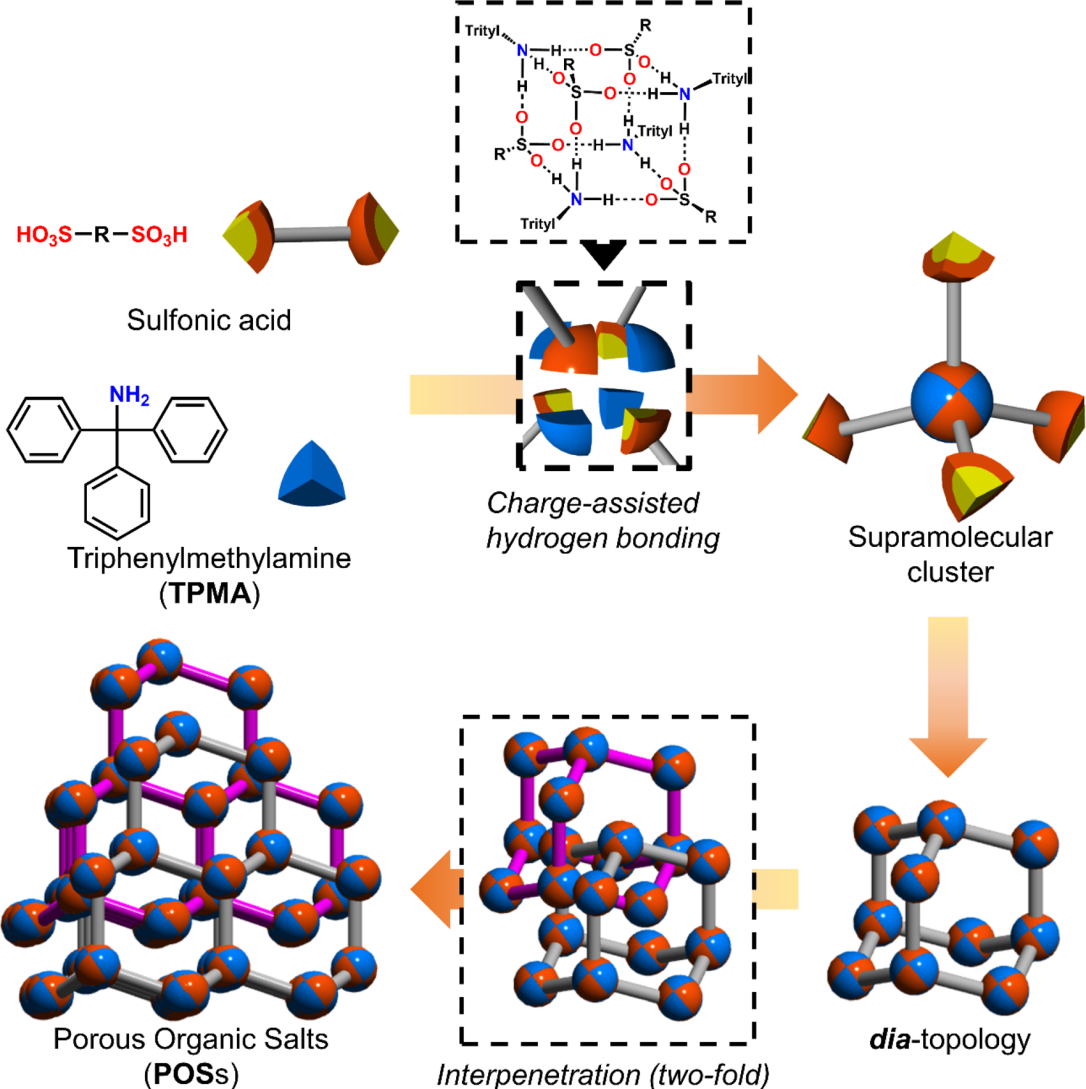
Schematic representation of the hierarchical construction of POSs with sulfonic acids and TPMA.

Among hydrogen bonds, the hydrogen bonds between strong acids such as sulfonic acids and bases such as amines, namely charge-assisted hydrogen bonds,^15^ have higher ionic character and binding energy than those of common hydrogen bonds. As shown in Fig. 1, we have reported that various sulfonic acids and bulky triphenylamine (TPMA) hierarchically construct porous organic salts (POSs).^16^ Four sulfo groups and four amino groups of TPMA form a tetrahedral [4 + 4] supramolecular cluster (Fig. 1 upper portion), and the clusters were connected to form diamondoid topology (***dia***-topology) (Fig. 1 right portion). Then, the frameworks were interpenetrated to construct a porous structure (Fig. 1 lower portion). Bulky trityl groups shielded sulfo groups and amino groups in the cluster from solvent molecules and prevented the involvement of solvent molecules in the charge-assisted hydrogen bonds (Fig. S1†),^17^ which enabled POSs surely to construct the porous structures composed of tetrahedral moieties. Recently, by using TPMA-X, where substituents (X) were introduced in the *para*-position of the benzene rings of TPMA, we successfully formed various environments in porous structures with ***dia***-topology by distortion of the framework and change of the style of interpenetration.^16*d*^ However, porous structures of POSs, *i.e.*, types of network topologies, have been limited to the ***dia***-topology.

**Fig. 2:**
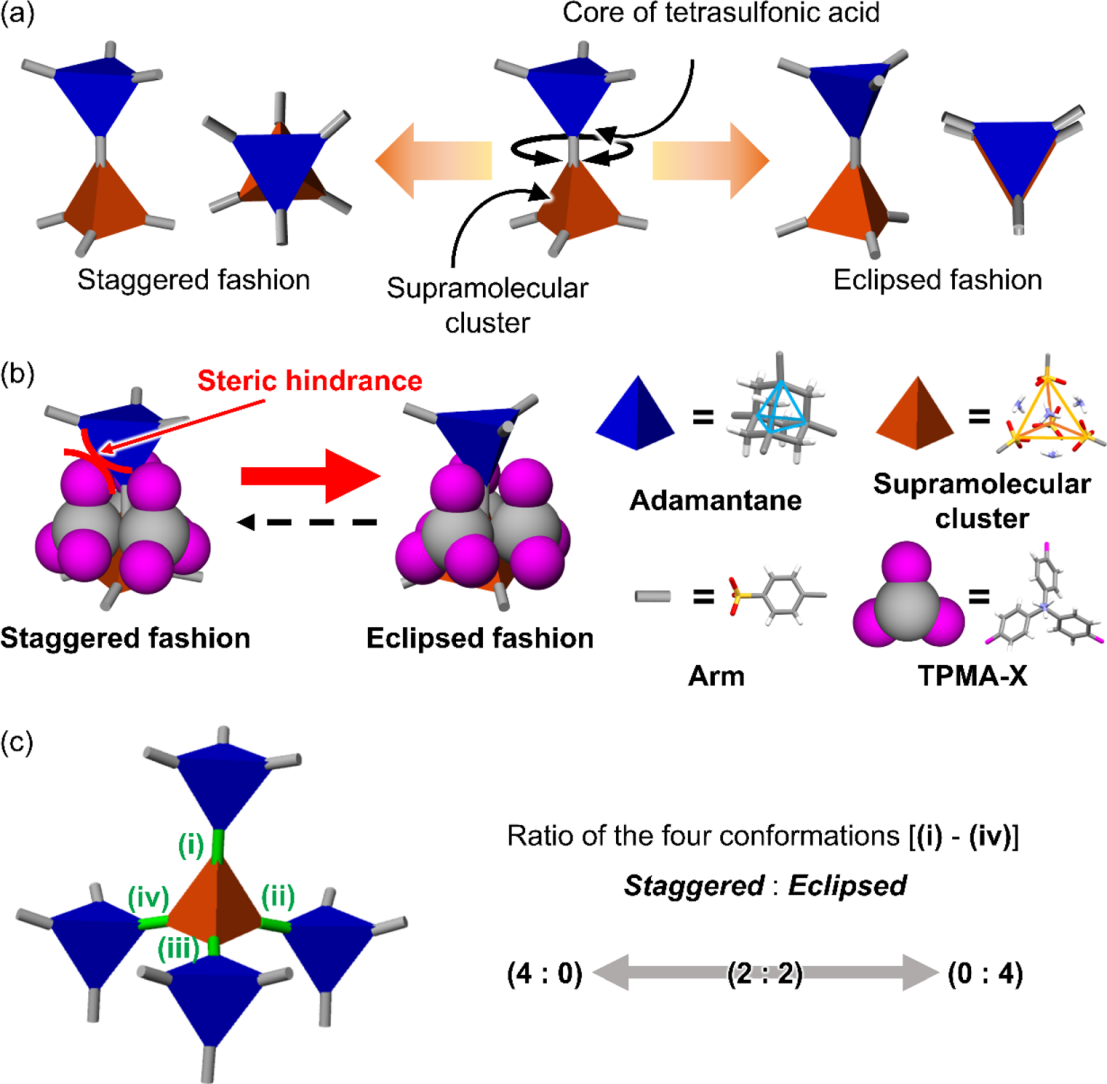
(a) Two connection fashions between the core of tetrasulfonic acid and the supramolecular cluster. (b) Conformation styles between the tetrahedron of the adamantane core (blue) of AdPS and the tetrahedron of the supramolecular cluster (orange), and the strategy to form eclipsed conformation by steric hindrance between the substituent X (pink) at TPMA-X and the adamantane core. (c) Schematic representations of the four conformations (green connection) between the four adamantane cores (blue) and a supramolecular cluster (orange) leading to the determination of the network topology of the porous structure.

**Scheme 1:**
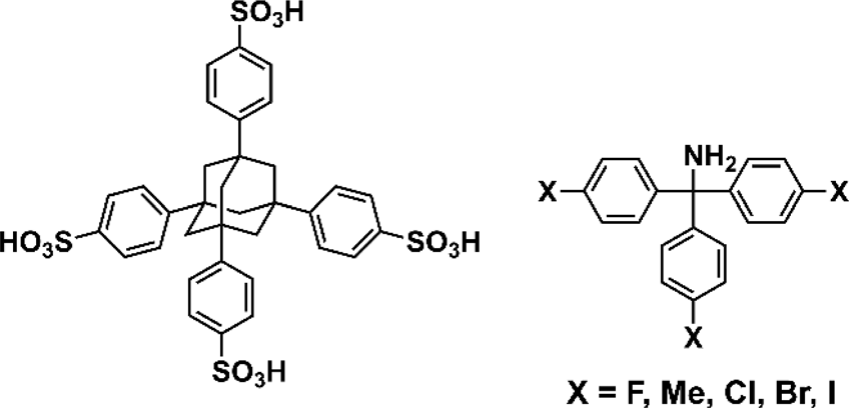
Chemical structures of porous organic salts.

Here, we aimed to control the network topology of HOFs toward diversification and functionalization of their porous structures. To form the different network topologies from the default network topology (***dia***-topology) in POSs, we focused on the conformation between connected two tetrahedral moieties (Fig. 2a), which are the core of tetrasulfonic acid (Fig. 2a blue tetrahedron, and Fig. 2b blue tetrahedron) and the supramolecular cluster (Fig. 2a orange tetrahedron, and Fig. 2b orange tetrahedron). We designed the POSs by combining tetrahedral-shaped tetrasulfonic acids with bulky adamantane (4,4′,4′′,4′′′-(adamantane-1,3,5,7-tetrayl) tetrabenzenesulfonic acid; AdPS) and TPMA-X (X = F, methyl (Me), Cl, Br, I) shown in Scheme 1, which provided the steric hindrance between adamantane (blue tetrahedron in Fig. 2b) and the substituents (X, purple ball in Fig. 2b) (as shown in Fig. 2b left portion), and we controlled the degree of steric hindrance by changing X. We changed the degree of steric hindrance and formed not only the common ***dia***-topology in POSs but also the rare network topology (***sod***-topology) and ***lon***- and ***uni***-topologies that are formed for the first time in HOFs. In the case of AdPS/TPMA-F and AdPS/TPMA-Me with a medium degree of steric hindrance, multiple network topologies were formed depending on the template molecules in recrystallization, and we successfully isolated each porous structure with only a specific network topology. While the three types of AdPS/TPMA-Me with different network topologies (***dia***-, ***lon***-, and ***sod***-topologies) were composed of the same components, they exhibited significantly different gas adsorption properties depending on their network topologies. AdPS/TPMA-Me with ***dia***-topology exhibited CO_2_, H_2_, and O_2_ adsorption and AdPS/TPMA-Me with ***lon***- and ***sod***-topologies exhibited selective CO_2_ adsorption.

## Results and discussion

POSs are composed of tetrahedral components (*e.g.*, core of tetrasulfonic acid (Fig. 2a blue tetrahedron) and a supramolecular cluster (Fig. 2a orange tetrahedron)). The style of conformation between two tetrahedral components (Fig. 2a blue and orange tetrahedra) includes staggered fashion and eclipsed fashion. Structures composed of tetrahedral components have the conformation with staggered fashion^18^ to form highly symmetrical^19^ and stable ***dia***-topology. In the case of POSs with tetrahedral-structured tetrasulfonic acid and TPMA (Fig. S2a†), two tetrahedra of the core of tetrasulfonic acid (Fig. S2b† blue tetrahedron) and the tetrahedral supramolecular cluster (Fig. S2b† orange tetrahedron) were linked in staggered fashion (Fig. S2b†) to form ***dia***-topology.^16*d*^ Therefore, to form the different network topologies from the default network topology (***dia***-topology), we aimed to change the style of conformation between the core of tetrasulfonic acid (Fig. 2b blue tetrahedron) and the supramolecular cluster (Fig. 2b orange tetrahedron) from staggered fashion to eclipsed fashion (Fig. 2b).

In the conformation with staggered fashion (Fig. 2a left portion), the three arms (Fig. 2a gray sticks on the blue tetrahedron) from a core of tetrasulfonic acid (Fig. 2a blue tetrahedron) and the three arms (Fig. 2a gray sticks on the orange tetrahedron) from a supramolecular cluster (Fig. 2a orange tetrahedron) were arranged in staggered fashion. Herein, POSs had two components such as sulfonic acids and amines. Trityl groups of TPMA (Fig. 2b gray ball and purple balls) were located on the faces of the tetrahedron of the supramolecular cluster (Fig. 2b orange tetrahedron). Furthermore, in one connection moiety between the core of tetrasulfonic acid and the supramolecular cluster (Fig. 2b left portion), three trityl groups (Fig. 2b gray ball and purple balls) existed between the core of tetrasulfonic acid (Fig. 2b blue tetrahedron) and the supramolecular cluster (Fig. 2b orange tetrahedron). Therefore, we hypothesized that the occurrence of steric hindrances between tetrasulfonic acids (Fig. 2b blue tetrahedron) and trityl groups (Fig. 2b gray ball and purple balls) allowed the tetrasulfonic acid and supramolecular cluster to form the conformation with eclipsed fashion (Fig. 2b center portion).

To provide the steric hindrance between tetrasulfonic acids and trityl groups (Fig. 2b left portion), we prepared the organic salts from AdPS and TPMA-X where substituents were introduced into TPMA (Scheme 1). Furthermore, one supramolecular cluster was connected to four tetrasulfonic acids (Fig. 2c left portion). Therefore, the whole network topology was determined by the style (staggered or eclipsed) and the ratio of the four conformations. Thus, to control the style and the ratio of the conformations and to form various network topologies, we used halogens and the methyl group (X = F, methyl (Me), Cl, Br, I) with different bulkiness (Scheme 1) as the substituents (X) of TPMA-X (Fig. 2b gray ball with three purple balls) and changed the degree of steric hindrance between tetrasulfonic acids (Fig. 2b blue tetrahedron) and trityl groups (Fig. 2b gray ball and purple balls) systematically.

**Fig. 3:**
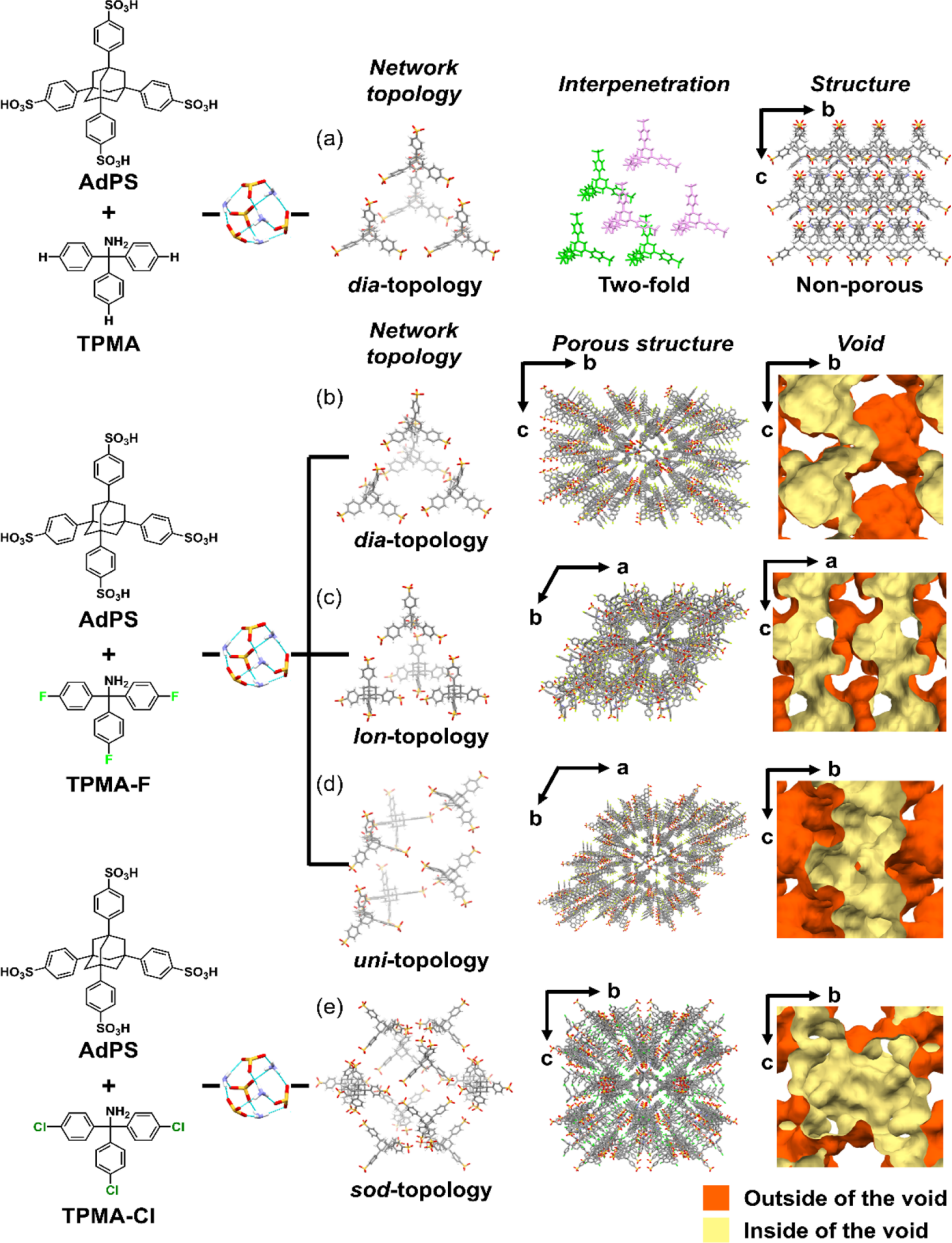
(a) Schematic representation of the resultant network topology, interpenetration, and the structure of AdPS/TPMA. Schematic representations of the resultant network topology, porous structure, and void structure of AdPS/TPMA-F with (b) ***dia***-topology, (c) ***lon***-topology, or (d) ***uni***-topology, and (e) AdPS/TPMA-Cl with ***sod***-topology.

In the organic salt of AdPS and TPMA with no substituents (AdPS/TPMA), the bulkiness of only adamantane was not enough to provide steric hindrance; therefore, only ***dia***-topology was formed (Fig. 3a Network topology). Furthermore, the frameworks with ***dia***-topology were interpenetrated to construct the non-porous structure (Fig. 3a Structure).

On the other hand, the organic salt of AdPS and TPMA-F where fluorine (F) was introduced into TPMA (AdPS/TPMA-F) formed ***dia***-topology without interpenetration by the bulkiness of the substituents (Fig. 3b Network topology). The porous structure of ***dia***-topology possessed the cage-like void (Fig. 3b Void) which was derived from the central void in the basic structure of ***dia***-topology composed of four AdPS (Fig. 3b Network topology), and the narrow bottleneck to connect the voids (Fig. 3b Void). The maximum and minimum pore sizes (Table S1†) calculated with the Poreblazer v4.0 (ref. 20) program that was commonly used in previous investigations,^21^ were 8.92 Å and 3.61 Å respectively. Furthermore, the use of different template molecules with different electron densities and molecular structures (see the ESI;† Preparation of the single-crystal of AdPS/TPMA-F), enabled us to form AdPS/TPMA-F with ***lon***-topology (Fig. 3c Network topology) and ***uni***-topology (Fig. 3d Network topology), respectively.

***lon***-topology is the framework of hexagonal diamond (Lonsdaleite),^22^ which is an allotrope of diamond. Therefore, to form ***lon***-topology, tetrahedral components are necessary, which is the same as the strategy for ***dia***-topology formation;^19^ therefore, ***lon***-topology could not be designed in HOFs. The porous structure of AdPS/TPMA-F with ***lon***-topology possessed the columnar void (Fig. 3c Void) which was derived from the hexagonal structure in ***lon***-topology, and the columnar voids were connected to form a three-dimensional void (Fig. 3c Void). The maximum and minimum pore sizes (Table S1†) calculated with the Poreblazer v4.0 (ref. 20) program were 8.37 Å and 4.96 Å respectively.

AdPS/TPMA-F with ***uni***-topology had a helical framework and a chiral structure with a three-fold helix (Fig. 3d Network topology), where three AdPS existed per turn of the helix (Fig. S3†). The porous structure possessed a triangular columnar void (Fig. 3d Void) which was derived from the three-fold helical structure. The maximum and minimum pore sizes (Table S1†) calculated with the Poreblazer v4.0 (ref. 20) program were 7.45 Å and 4.84 Å respectively. Chiral HOFs have been often constructed using chiral building blocks as a component, *i.e.*, the chirality of the building blocks has a direct effect.^23^ It should be noted that even though the helical structure of AdPS/TPMA-F with ***uni***-topology was constructed from achiral building blocks, using *R*- or *S*-form of the chiral template molecules (*e.g.*, carvone) in recrystallization enabled to us induce the corresponding chiralities of the helix, respectively. The *R*-form of carvone was used to form the left-handed helical structure (Fig. S3a and b†) and the *S*-form of carvone was used to form the right-handed helical structure (Fig. S3c and d†).

Next, the organic salt of AdPS and TPMA-Cl whose substituents (X) were bulkier than that of TPMA-F (AdPS/TPMA-Cl), formed ***sod***-topology which was the same network topology as sodalite (Fig. 3e Network topology). Even by changing the template molecules, AdPS/TPMA-Cl formed only ***sod***-topology. The maximum and minimum pore sizes (Table S2†) calculated with the Poreblazer v4.0 (ref. 20) program were 15.7 Å and 5.88 Å respectively. The basic structure of the ***sod***-topology of AdPS/TPMA-Cl was composed of twelve AdPS (Fig. 3e Network topology) and formed a larger void (Fig. 3e Void) than those of the other network topologies. Generally, bulkier substituents decrease the pore size.^16*d*,24^ Although the maximum pore size of AdPS/TPMA-F with ***dia***-topology, ***lon***-topology, and ***uni***-topology was 8.92 Å, 8.37 Å, and 7.45 Å respectively (Table S1†), the maximum pore size of AdPS/TPMA-Cl with ***sod***-topology was 15.7 Å (Table S2†) because of the formation of ***sod***-topology and large voids.

**Fig. 4:**
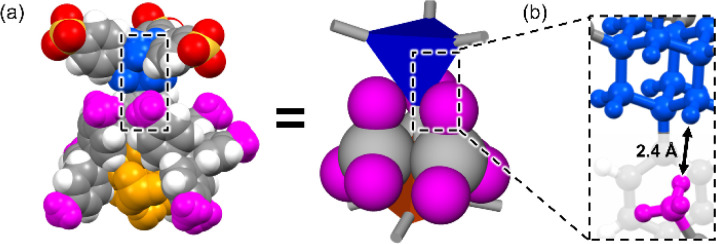
(a) Corey–Pauling–Koltun (CPK) molecular model and schematic representation of staggered conformation between the adamantane core (blue) and supramolecular cluster (orange) of AdPS/TPMA-Me with ***dia***-topology. (b) Distance between the hydrogen of adamantane of AdPS and hydrogen of the methyl group (pink) of TPMA-Me.

Powder X-ray diffraction (PXRD) patterns of AdPS/TPMA-X were measured (Fig. S4–S6†). Characteristic PXRD peaks (2*θ* = 6.22° (AdPS/TPMA-F with ***dia***-topology), 5.76°, 6.06°, and 6.50° (AdPS/TPMA-F with ***lon***-topology), 3.74°, and 6.36° (AdPS/TPMA-F with ***uni***-topology), 6.08° (AdPS/TPMA-Me with ***dia***-topology), 5.66°, 6.08°, and 6.48° (AdPS/TPMA-Me with ***lon***-topology), 3.90° (AdPS/TPMA-Me with ***sod***-topology), 4.08° (AdPS/TPMA-Cl with ***sod***-topology), 4.04° (AdPS/TPMA-Br with ***sod***-topology), 4.12° (AdPS/TPMA-I with ***sod***-topology)) in the low angle region of immediately following formation *via* crystallization were identical to those of simulated patterns from the crystal structures, respectively. The previous investigations^25^ of PXRD patterns of porous materials indicated that differences in the higher angle region (2*θ* > 10°) were attributed to the existence of molecules in the pore, and identification of characteristic peaks in the lower angle region meant that structures were the same. Therefore, in our case, the structures formed *via* crystallization would be identical to their crystal structures, which indicated that AdPS/TPMA-X with different network topologies was isolated respectively. These obtained crystals were soluble in methanol and decomposable into their components, which indicated the high chemical recyclability of AdPS/TPMA-X. We summarized the obtained network topologies in order of the bulkiness of substituents (X) in Table 1. The organic salts that formed ***dia***-topology were limited up to AdPS/TPMA-Me (Table 1). To investigate the reason for the topology change, we focused on the crystal structure of AdPS/TPMA-Me with ***dia***-topology (Fig. 4a). As shown in Fig. 4b, in the structure of ***dia***-topology, the methyl group of TPMA-Me was located close to the methylene moiety of adamantane, and the distance between the hydrogen atom of the methyl group and the hydrogen atom of adamantane was 2.4 Å (Fig. 4b). This distance was equal to the sum of the van der Waals radius of two hydrogen atoms (1.2 Å),^26^ which indicated the contact of the methyl group and the methylene moiety of adamantane. Therefore, when the substituent was bulkier than the methyl group, the conformation with staggered fashion could not be formed by steric hindrances, and the conformation was considered to change from staggered fashion to eclipsed fashion.

**Table 1:** Obtained network topologies of AdPS/TPMA-X

AdPS/TPMA-X	Radius of X (Å)^27^	Topology			
AdPS/TPMA	1.20	***dia*** (two-fold)	*lon*	*uni*	*sod*
AdPS/TPMA-F	1.47	***dia***	***lon***	***uni***	*sod*
AdPS/TPMA-Me	1.58^a^	***dia***	***lon***	*uni*	***sod***
AdPS/TPMA-Cl	1.75	*dia*	*lon*	*uni*	***sod***
AdPS/TPMA-Br	1.85	*dia*	*lon*	*uni*	***sod***
AdPS/TPMA-I	1.98	*dia*	*lon*	*uni*	***sod***

a van der Waals radius parallel to the group axis.

**Fig. 5:**
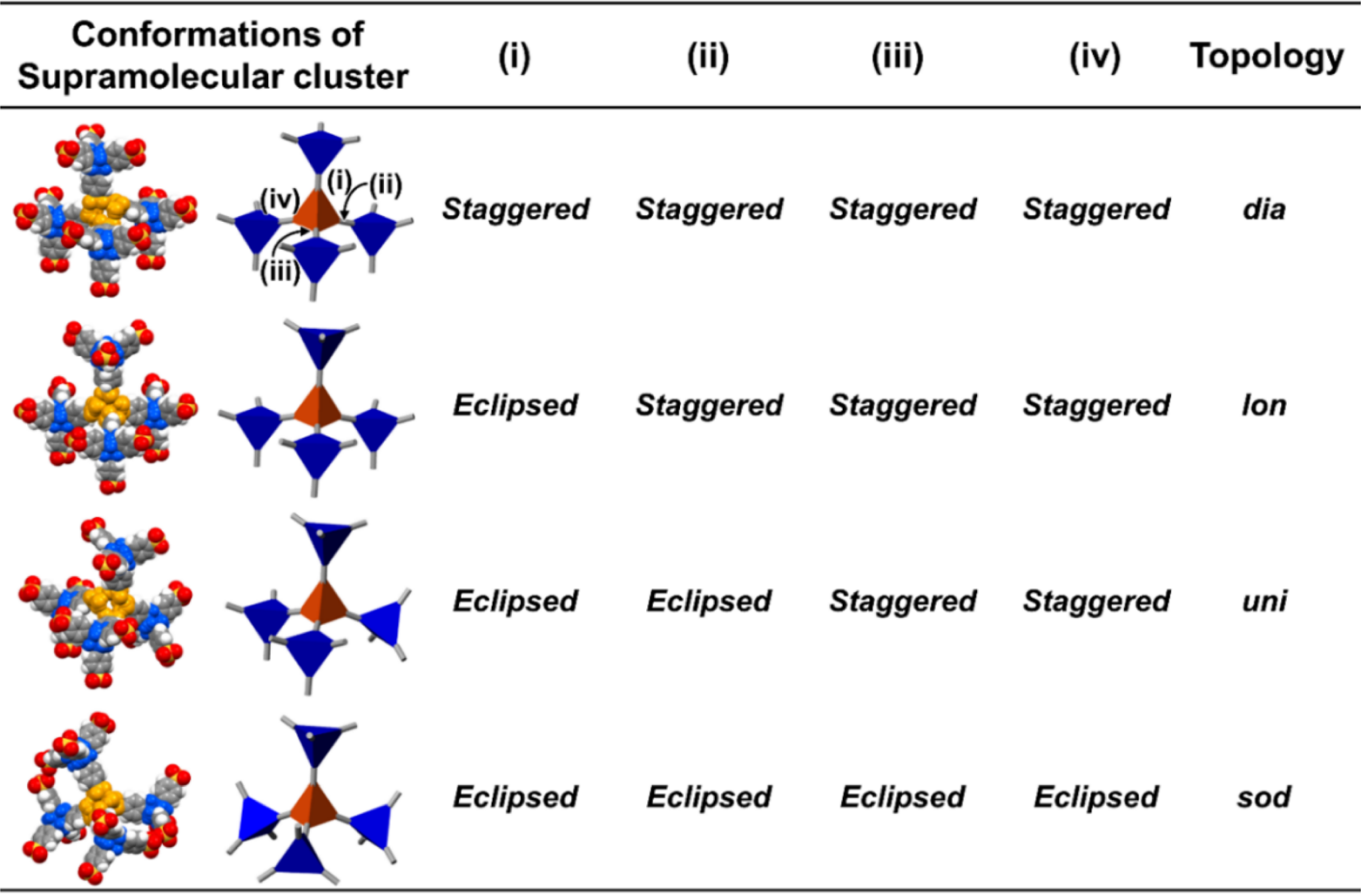
Corey–Pauling–Koltun (CPK) molecular model and schematic representations of the four conformations between the four adamantane cores (blue) and a supramolecular cluster (orange) in ***dia***-topology, ***lon***-topology, ***uni***-topology, and ***sod***-topology.

As shown in Fig. 2c left portion, one supramolecular cluster had four conformations, and the bulkier substituents changed the conformation from staggered fashion to eclipsed fashion. From the conformation between the core of tetrasulfonic acid (Fig. 5 blue tetrahedron) and the supramolecular cluster (Fig. 5 orange tetrahedron) based on the crystal structures (Fig. 5 left portion of first column) of the obtained four-type network topologies, the ratios of the conformations (staggered fashion *versus* eclipsed fashion) of each network topology were summarized in Fig. 5. When the styles of conformations were all staggered fashion, ***dia***-topology was formed (Fig. 5***dia***-topology). However, when the style of one conformation was eclipsed fashion and that of other conformations was staggered fashion in the four conformations, ***lon***-topology was formed (Fig. 5***lon***-topology). Then, when the style of two conformations was staggered fashion and that of other conformations was eclipsed fashion in the four conformations, ***uni***-topology was formed (Fig. 5***uni***-topology), and when the styles of conformations were all eclipsed fashion, ***sod***-topology was formed (Fig. 5***sod***-topology). Therefore, as the substituents were bulkier, the formation possibility of network topology increased in the order of ***dia***-topology < ***lon***-topology < ***uni***-topology < ***sod***-topology. In fact, as shown in Table 1, AdPS/TPMA with the lowest degree of steric hindrance formed only ***dia***-topology where the style of all conformations was staggered fashion. AdPS/TPMA-F and AdPS/TPMA-Me had a higher degree of steric hindrance than AdPS/TPMA and formed not only ***dia***-topology but also ***lon***- and ***uni***-topologies where the style of one or two conformations among the four was eclipsed fashion. Furthermore, AdPS/TPMA-Cl, AdPS/TPMA-Br, and AdPS/TPMA-I had the bulkier substituents than the methyl group, provided a high degree of steric hindrance, and formed only ***sod***-topology where the style of all conformations was eclipsed fashion. In addition, to remove the difference of the condition in recrystallization, PXRD measurements of AdPS/TPMA-X (X= F, Me, Cl, Br, I), which was prepared under the same conditions (template molecule: 1-methylnaphthalene, solvent: methanol, temperature: 25 °C), were performed. AdPS/TPMA-X (X= Me, Cl, Br, I) had ***sod***-topology but AdPS/TPMA-F had ***lon***-topology (Fig. S7†), which supported that the network topologies with potential for formation were determined not by recrystallization conditions but by the degree of steric hindrance.

According to Table 1, AdPS/TPMA-F and AdPS/TPMA-Me had a medium degree of steric hindrance, which enabled them to form multiple network topologies depending on the template molecules. For example, in the case of AdPS/TPMA-Me, when the template molecule was altered from (−)-β-pinene without aromatic rings to benzonitrile with an aromatic ring, the resultant network topology was changed from ***dia***-topology to ***sod***-topology (see the ESI;† Preparation of the single-crystal of AdPS/TPMA-Me). This was presumably because the template molecules which had strong interaction (*e.g.*, π–π interaction) with the benzene rings in TPMA-X, predominately located close to TPMA-X, which increased the degree of steric hindrance surrounding a supramolecular cluster.

The difference in network topology is known to change the pore sizes of porous structures and even the gas adsorption properties.^9*c*,*e*,28^ To investigate the pure effect of the different network topologies on their gas adsorption properties, we used AdPS/TPMA-Me which had methyl groups with few interactions for gases and significantly different three-type network topologies (***dia***-, ***lon***-, and ***sod***-topologies). The porous structures of AdPS/TPMA-Me with ***dia***-topology (Fig. S8a†) possessed the cage-like void (Fig. S8d†), and the maximum and minimum pore sizes (Table S3†) calculated with the Poreblazer v4.0 (ref. 20) program were 6.25 Å and 3.66 Å respectively. The porous structures of AdPS/TPMA-Me with ***lon***-topology (Fig. S8b†) possessed the columnar void (Fig. S8e†), and the maximum and minimum pore sizes (Table S3†) calculated with the Poreblazer v4.0 (ref. 20) program were 7.06 Å and 5.65 Å respectively. The porous structures of AdPS/TPMA-Me with ***sod***-topology (Fig. S8c†) possessed the cage-like void (Fig. S8f†), and the maximum and minimum pore sizes (Table S3†) calculated with the Poreblazer v4.0 (ref. 20) program were 15.6 Å and 4.57 Å respectively.

To perform gas adsorption measurements, we activated the porous structures of AdPS/TPMA-Me with ***dia***-, ***lon***-, and ***sod***-topologies. AdPS/TPMA-Me with ***dia***-topology was successfully activated by drying at 80 °C. However, in the case of AdPS/TPMA-Me with ***lon***-topology and ***sod***-topology, the template molecules were not completely removed by drying. Therefore, they were activated using supercritical CO_2_ fluid.

FT-IR measurements of the activated AdPS/TPMA-Me with ***dia***-, ***lon***-, and ***sod***-topologies did not show NH stretching mode (3361 and 3296 cm^−1^) of TPMA-Me (Fig. S9†). In addition, S=O stretching mode of the activated AdPS/TPMA-Me with ***dia***-, ***lon***-, and ***sod***-topologies (1163, 1124, 1034, and 1007 cm^−1^) was blue shifted from that of AdPS (1123, 1032, 1003 cm^−1^), which indicated that protons of sulfo groups were captured by hydrogen bond acceptors. Therefore, these results indicated that all sulfo groups and amino groups formed charge-assisted hydrogen bonds (Fig. S9†). Furthermore, elemental analyses revealed that AdPS and TPMA-Me were in the ratio of 1 to 4 (see the ESI;† Element analyses of AdPS/TPMA-Me with ***dia***, ***lon***, and ***sod***-topologies). These results indicated that supramolecular clusters were not damaged and amorphous impurities were not formed during activation.

PXRD measurements of activated AdPS/TPMA-Me with ***dia***-, ***lon***-, and ***sod***-topologies were also performed (Fig. S5†). In PXRD patterns after activation (Fig. S5a and c†), porous structures with ***dia***-topology and ***sod***-topology were not changed by activation. However, in the case of the porous structure with ***lon***-topology, the wide-angle shift of the diffraction peaks by activation was observed (Fig. S5b†). The diffraction peak at 5.7° of the porous structure with ***lon***-topology immediately after recrystallization corresponded to the (100) and (001) planes, and the diffraction peak at 6.5° corresponded to the (011) and (110) planes (Fig. S5b†). In the porous structure after activation, the former peak shifted to 6.4° and the latter shifted to 7.2°.

The peak shapes of AdPS/TPMA-Me with ***lon***-topology before and after activation (Fig. S5b†) were similar; FT-IR spectra (Fig. S9†) indicated that the supramolecular clusters connecting AdPS and TPMA-Me were not damaged, and peak locations of FT-IR spectra before and after activation (Fig. S10†) did not have much difference; therefore, the porous structure would shrink by 1.4–1.8 Å along three directions while maintaining ***lon***-topology.

To investigate the retainment of the network topology in detail, we performed variable-temperature (VT) PXRD (Fig. S11a†). VT-PXRD of the porous structure with ***lon***-topology showed the wide-angle shifts of the diffraction peaks in the range of 70 °C to 119 °C, and these shifts had the same trend as those of ***lon***-topology after activation. In addition to thermogravimetric analysis (TGA) results (Fig. S11c†), these indicated structural transition following the removal of template molecules. Furthermore, from 119 °C, the low-angle shifts of diffraction peaks occurred, and the peak returned in the original peak location of the porous structure with ***lon***-topology at 194 °C (Fig. S11b†). From these results it can be inferred that the removal of template molecules induced the structure with ***lon***-topology to shrink, but the network topology was maintained up to 194 °C and also after activation.

**Fig. 6:**
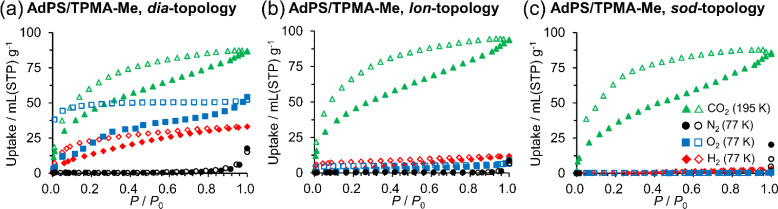
Gas adsorption isotherms of ***dia***-topology (a), ***lon***-topology (b), and ***sod***-topology (c) of AdPS/TPMA-Me: CO_2_ (195 K), N_2_ (77 K), O_2_ (77 K), H_2_ (77 K). Filled symbols: adsorption process, open symbols: desorption process. *P* denotes the pressure at adsorption and *P*_0_ denotes the atmospheric pressure.

We performed CO_2_, N_2_, O_2_, and H_2_ gas adsorption measurements of the activated porous materials at 195 K, 77 K, 77 K, and 77 K, respectively. Adsorption isotherms of AdPS/TPMA-Me with different network topologies are shown in Fig. 6a–c, respectively. All porous structures of AdPS/TPMA-Me adsorbed more CO_2_ than the other gas, and this adsorption trend was also observed in the adsorption isotherms of CO_2_, N_2_, and O_2_ at 195 K (Fig. S12†), which exhibited a higher affinity for CO_2_. This was presumably due to the following three reasons: (1) the smaller kinetic diameter of CO_2_ (3.30 Å (ref. 29)) than those of N_2_ (3.80 Å (ref. 29)) and O_2_ (3.46 Å (ref. 29)), (2) the quadrupole–quadrupole interaction^30^ between CO_2_ and benzene rings^30^ with a large quadrupole moment, and (3) the interaction^31^ between CO_2_ and polar moieties of the porous structure that was composed of sulfonic acid and amines.

**Fig. 7:**
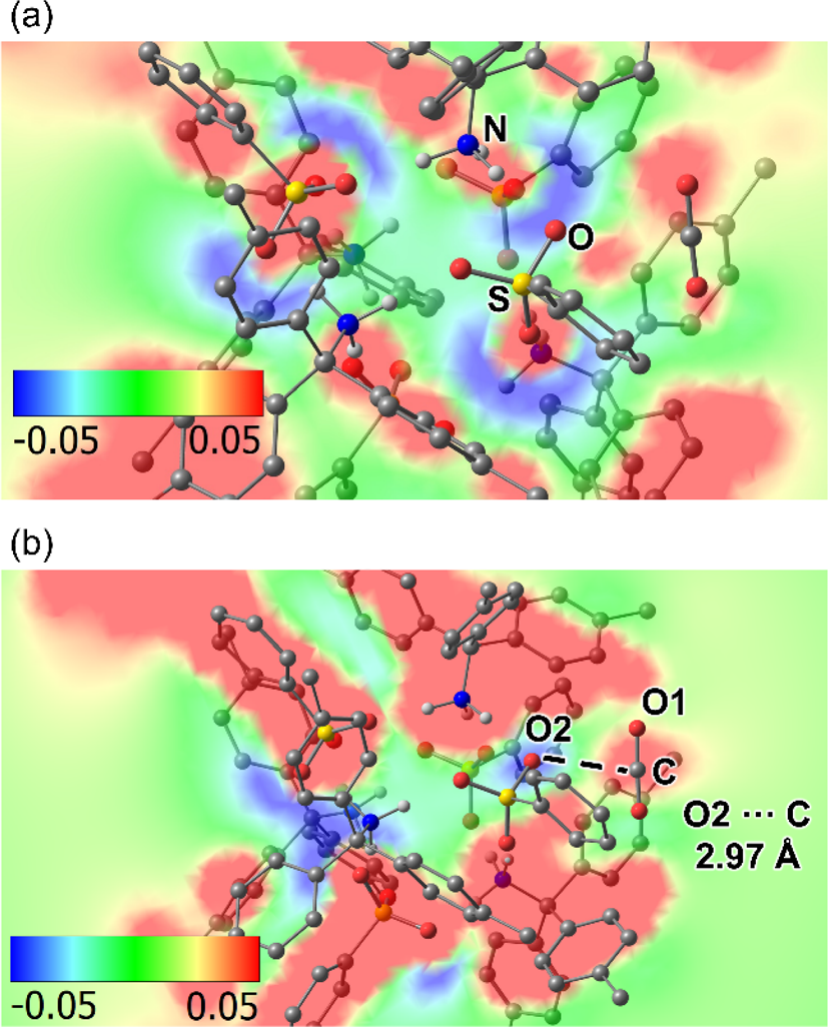
The electrostatic potential map of the supramolecular cluster in AdPS/TPMA-Me with ***dia***-topology and a CO_2_: (a) the plane composed of S, O, and N. (b) The plane composed of C, O_1_, and O_2_.

To confirm this hypothesis (3), density functional theory (DFT) calculation of a supramolecular cluster in AdPS/TPMA-Me with ***dia***-topology and a CO_2_ molecule was performed (Fig. S13†). As shown in Fig. 7a, the electrostatic potential map of the plane composed of a sulfur atom (S) and an oxygen atom (O) in a sulfo group, and a nitrogen atom (N) in an amino group showed that oxygen atoms of sulfo groups were negatively charged and amino groups were positively charged, which indicated that the polar moieties existed in the supramolecular clusters. Furthermore, as shown in Fig. 7b, the electrostatic potential map of the plane composed of a carbon atom (C) and an oxygen atom (O_1_) in the CO_2_, and an oxygen atom (O_2_) in the sulfo group showed that O_2_ was negatively charged and C was positively charged, and the distance between O_2_ and C was 2.97 Å. These results indicated that oxygen atoms in sulfo groups interacted with CO_2_. The calculated adsorption energy (−45.98 kJ mol^−1^) located in a strong physisorption range (30–50 kJ mol^−1^),^32^ which supported that AdPS/TPMA-Me with polar moieties had a higher affinity for CO_2_.

As depicted in Fig. 6b and c, the porous structures with ***lon***-topology and ***sod***-topology selectively adsorbed CO_2_, and their CO_2_ isotherms showed hysteresises. This should be attributed to strong interaction between CO_2_ and the polar moieties on their pore surfaces. The previous investigations^31*b*,33^ indicated that pore surfaces with polarity cannot provide sufficient energy of adsorption for non-polar gas molecules, and the porous materials with polarity did not adsorb them. In our case, supramolecular clusters with the polar moiety existed in the pore surfaces of AdPS/TPMA-Me with not only ***dia***-topology (Fig. 7) but also ***lon***-topology and ***sod***-topology; therefore, based on the effect of polarity of the supramolecular cluster, these porous materials did not adsorb N_2_, O_2_, and H_2_. In order to investigate the influence of the substituents (X) on gas adsorption properties, the gas adsorption measurement of AdPS/TPMA-F with ***lon***-topology was performed. In the case of the same ***lon***-topology, despite the difference of substituents (X), AdPS/TPMA-F with ***lon***-topology showed similar gas adsorption properties (Fig. S14†) to that of AdPS/TPMA-Me with ***lon***-topology. The CO_2_ isotherm of AdPS/TPMA-F with ***lon***-topology had larger hysteresis than that of AdPS/TPMA-Me with ***lon***-topology (Fig. 6b and S14†), which indicated that the high electronegativity of fluorine atoms^34^ increased the interaction between CO_2_ and the substituent (X = F) on the pore surface.

As depicted in Fig. 6a, AdPS/TPMA-Me with ***dia***-topology adsorbed not only CO_2_ but also H_2_ and O_2_. Furthermore, hystereses were observed in isotherms of CO_2_, H_2_, and O_2_, which suggested that they were attributed to not only the interaction between CO_2_ and the polar moieties but also the narrow bottleneck. Despite the effect of the polar pore, the porous structure with ***dia***-topology adsorbed non-polar H_2_ and O_2_. This might be because the size of the bottleneck (3.66 Å (Table S3†)) was similar to that of O_2_ (3.46 Å (ref. 29)) and H_2_ (2.89 Å (ref. 29)), which induced the van der Waals forces with gas molecules to get stronger.^24,26*a*,35^ Therefore, the van der Waals forces overcome the effect of the polar pore, and the porous structure with ***dia***-topology also adsorbed O_2_ and H_2_. While O_2_ and H_2_ were adsorbed at 77 K, N_2_ was not adsorbed (Fig. 6a). This was presumably because N_2_ (3.80 Å (ref. 29)) with a larger kinetic diameter than the size of the bottleneck (3.66 Å (Table S3†)) did not pass through the bottleneck.

Finally, Kr adsorption measurements of AdPS/TPMA-Me with ***dia***-, ***lon***-, and ***sod***-topologies at 77 K were conducted to calculate the Brunauer–Emmett–Teller (BET) surface areas and pore volumes (Fig. S15†). The surface area analysis with Kr has higher sensitivity than those with N_2_ and Ar.^36^ However, AdPS/TPMA-Me with ***dia***-, ***lon***-, and ***sod***-topologies did not adsorb Kr (3.66 Å (ref. 29)) presumably due to the narrow bottleneck (3.66 Å (Table S3†) in ***dia***-topology) and the effect of the polar pore. In previous investigation,^37^ BET surface area of HOFs with no adsorption for N_2_ was calculated based on the CO_2_ isotherm; therefore, in the case of AdPS/TPMA-Me, BET surface areas and pore volumes were calculated based on the CO_2_ isotherms to be 228 m^2^ g^−1^ and 0.236 cm^3^ g^−1^ in the porous structure with ***dia***-topology, 228 m^2^ g^−1^ and 0.255 cm^3^ g^−1^ in the porous structure with ***lon***-topology, and 194 m^2^ g^−1^ and 0.231 cm^3^ g^−1^ in the porous structure with ***sod***-topology respectively (Table S4†).

We revealed that despite the same components, the difference in network topologies enables a change in gas adsorption properties dramatically.

## Conclusions

We focused on the steric hindrance between tetrasulfonic acid with a bulky adamantane core (AdPS) and TPMA-X. By changing the substituents (X) in TPMA-X, we changed the style of conformation between two tetrahedra (adamantane and supramolecular cluster) from staggered fashion to eclipsed fashion. Therefore, the four-type network topologies (***dia***-, ***lon***-, ***uni***-, and ***sod***-topologies) were successfully formed and isolated. ***lon***- and ***uni***-topologies were obtained for the first time in HOFs. AdPS/TPMA-Me had a medium degree of steric hindrance, which enabled the formation of three-type network topologies (***dia***-, ***lon***-, and ***sod***-topologies); therefore, the change of template molecules used in recrystallization helped in successfully isolating these network topologies facilely. Further analyses of the relationship between network topologies, substituents, and recrystallization conditions will be performed in our future work. Despite the same components, AdPS/TPMA-Me with different network topologies exhibited significantly different gas adsorption properties. Based on these gas adsorption properties, AdPS/TPMA-Me with ***dia***-, ***lon***-, and ***sod***-topologies is expected for applications such as the generation of high-purity oxygen and the separation of CO_2_ from air. In our future work, we will investigate the relationship between substituents, network topologies, and gas adsorption properties. We showed that changing the conformation style of components determined the network topologies that are difficult to form or have not been formed in HOFs and achieved the proof-of-concept for the formation, diversification, and control of the network topologies and concomitant functions of HOFs. Furthermore, we revealed the relationship between ***lon***- and ***uni***-topologies and the conformation style and ratio. Therefore, this investigation leads to structural diversification and functionalization of HOFs, COFs, and metal–organic frameworks.

## Data availability

Details of materials, instruments, experimental procedures, crystallographic, and DFT calculations are given in the ESI† for this manuscript.

## Author contributions

Hiroi Sei: conceptualization, methodology, formal analysis, data curation, investigation, and writing – original draft. Kouki Oka: conceptualization, methodology, formal analysis, data curation, project administration, validation, supervision, and writing – original draft. Yuta Hori: software, formal analysis, and writing – review & editing. Yasuteru Shigeta: software, formal analysis, and writing – review & editing. Norimitsu Tohnai: conceptualization, methodology, data curation, project administration, validation, supervision, writing – review & editing, funding acquisition, and resources.

## Conflicts of interest

There are no conflicts of interest to declare.

## Supplementary Material

SC-015-D4SC01218F-s001

SC-015-D4SC01218F-s002
