# Supplementary material for: Network topology diversification of porous organic salts
Source: Chem Sci. 2024 May 1;15(21):8008–18. doi: 10.1039/d4sc01218f (PMC11134405; doi:10.1039/d4sc01218f)
Supplement: SC-015-D4SC01218F-s001 [file SC-015-D4SC01218F-s001.pdf]

Electronic Supplementary Material (ESI) for Chemical Science.
This journal is © The Royal Society of Chemistry 2024

Network Topology Diversification of Porous Organic Salts

Hiroi Sei,[ab†] Kouki Oka,[ab†] Yuta Hori,[c] Yasuteru Shigeta,[c] and Norimitsu Tohnai*[a]

a. Department of Applied Chemistry, Graduate School of Engineering, Osaka University, 2-1 Yamadaoka, Suita, Osaka 565-0871, Japan. Email: tohnai@chem.eng.osaka-u.ac.jp (Norimitsu Tohnai)

b. Institute of Multidisciplinary Research for Advanced Materials, Tohoku University, 2-1-1 Katahira, Aoba-ku, Sendai, Miyagi 980-8577, Japan.

c. Center for Computational Sciences, University of Tsukuba, 1-1-1 Tennodai, Tsukuba, Ibaraki 305-8577, Japan.

[†]H.S. and K.O. contributed equally to this work.

1. Experimental sections

Materials.

Magnesium (turnings) was purchased from Nacalai Tesque, Inc. Iodine was purchased from Sigma-Aldrich Co. LCC. Potassium iodide was purchased from Kanto Chemical Co., Inc. 1-bromoadamantane, 4-bromochlorobenzene, 4-bromotoluene, (-)-β-pinene, (+)-3-carene, (R)-(-)-carvone, (S)-(+)-carvone, 1,4-dibromobenzene, 4,4'-dichlorobenzophenone, *t*-butyl bromide, *n*-butyllithium, pararosaniline hydrochloride, thionyl chloride, tris(4-fluorophenyl)bromomethane, *N*,*N*-dimethylacetamide, triphenyl methylamine, and 1-methylnaphthalene were purchased from Tokyo Chemical Industry Co., Ltd. Aluminum chloride anhydrous, sodium nitrite, ammonium chloride, dioxane, sulfuric acid (97%), hydrochloric acid (35%) and nitrobenzene were purchased from Kishida Chemical Co., Ltd. Other chemicals were purchased from FUJIFILM Wako Pure Chemical Corporation.

Measurements.

Proton and carbon nuclear magnetic resonance (^{1}H NMR and ^{13}C NMR) spectra were recorded by a JEOL 400 JJYH (400 MHz) spectrometer with chemical shifts downfield from tetramethylsilane as the internal standard.

Thermal analyses were performed with a Rigaku ThermoPlus EVO2 TG8121 at a heating rate of 3 °C min^{-1} under nitrogen.

Gas adsorption measurements were performed on BELSORP-max from MicrotracBEL, Japan. The adsorption isotherms for N_2, O_2, and H_2 were corrected at 77 K. The adsorption isotherm for CO_2 was corrected at 195 K. Before all measurements, the samples were dried under reduced pressure and 353 K for 3 h.

The X-ray diffraction data of the organic salts was collected on a two-dimensional X-ray detector (PILATUS 200 K/R) equipped in Rigaku XtaLAB PRO diffractometer using thin multi-layer mirror monochromated Cu-Kα radiation (λ = 1.54187 Å). The cell refinements were performed with CrysAlisPro a software 1.171.39.5. SHELXT was used for the structure solution of the crystals. All calculations were performed with the observed reflections [I>2σ(I)] with the program CrystalStructure crystallographic software packages, except for refinement which was performed by SHELXL. All non-hydrogen atoms, except for highly disordered solvent molecules accommodated in voids, were refined with anisotropic displacement parameters, and hydrogen atoms were placed in idealized positions and refined as rigid atoms with the relative isotropic displacement parameters. SQUEEZE function equipped in the PLATON program was used to remove severely disordered solvent molecules in the voids for the porous organic salts (POSs).

Powder X-ray diffraction (PXRD) was performed with a Rigaku Ultima IV using graphite monochromatized Cu-Kα radiation (λ = 1.54187 Å) at 25°C.

POSs placed on an aluminum substrate were subjected to variable temperature (VT)-PXRD measurement under the air atmosphere. PXRD data was performed with a Rigaku Ultima-IV using graphite-monochromatized Cu-Kα radiation (λ = 1.54187 Å) with a temperature control unit. The temperature of the sample was increased from 25°C to 360°C with a heating rate of 1.0°C/min. As temperature increases, PXRD patterns ranging from 3° to 20.8° were repeatedly recorded at a scan rate of 3°/min. The temperature width of each PXRD scan was 6.0°C.

FT-IR spectra were recorded on a JASCO FT/IR-4200 FT-IR spectrometer.

Elemental analysis for C, H, and N elements was performed using MICRO CORDER JM 10 from J-SCIENCE Lab. Elemental analysis for S element was performed using organic elements analysis system XS-2100H (Mitsubishi Chemical Analytech). Prior to measurements, **AdPS/TPMA-Me** with ***dia***-, ***lon***-, and ***sod***-topologies have been activated.

Topological Analyses.

Topological analyses of **AdPS/TPMA-X** (**X= H, F, Me, Cl, Br, I**) were performed by ToposPro program package.[1] To consider that **AdPS/TPMA-X** were connected intermolecular bonds (i.e. Hydrogen bonds), we changed bond types of their hydrogen bonds from H-bond to valence and then, the simplification of their framework was performed by the standard method in the program. Their topological types were determined after simplification.

Calculations.

Density functional theory (DFT) calculations were performed for a supramolecular cluster and a CO_2 molecule, in which the B3LYP functional with the 6-31G(d) basis set was used. Grimme's D3 method was used to account for dispersion correction.[2] All DFT calculations were performed by the Gaussian 16 package.[3] The calculation model was constructed by extracting the part of **AdPS/TPMA-Me** with ***dia***-topology as a supramolecular cluster model (Figure S13). The geometrical optimization was then carried out for only hydrogen and CO_2 positions.

Synthesis of tris(4-fluorophenyl)methylamine (TPMA-F).

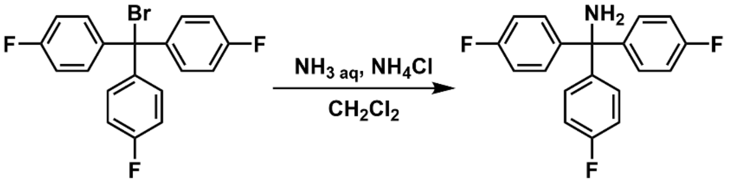

Scheme S1. Synthesis of tris(4-fluorophenyl)methylamine.

We synthesized tris(4-fluorophenyl)methylamine following the previous paper.[4] Tris(4-fluorophenyl)bromomethane (1.00 g, 2.65 mmol) and dichloromethane (80 mL) were added to aqueous ammonia solution (30 wt. %, 30 mL) containing ammonium chloride (1.40 g, 26.2 mmol) at 0°C, and the mixture was stirred for 6 h at 25°C. Then, the reaction was quenched by adding aqueous $Na_2S_2O_5$ solution. The mixture was extracted with dichloromethane/water, and then was purified using column chromatography on silica gel with dichloromethane as an eluent to give a pale-yellow solid. The solid was characterized as **TPMA-F**[4] (651 mg, 78 %), as follows (Figures S16 and S17): ^{1}H NMR (400 MHz, chloroform-d_1, δ): 7.22 (dd, J = 8.8 Hz, 6H), 7.04 (t, J = 8.8 Hz, 6H); ^{13}C NMR (400 MHz, chloroform-d_1, δ): 161.6, 144.1, 129.6, 114.8, 65.1.

Synthesis of tri-p-tolylmethanamine (TPMA-Me).

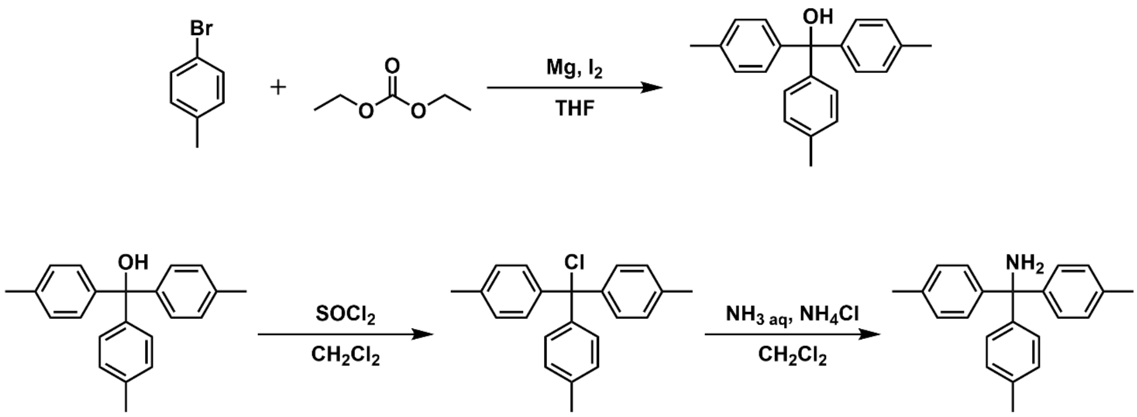

Scheme S2. Synthesis of tri-*p*-tolylmethanamine.

Magnesium (0.702 g, 28.9 mmol) and iodine (1 piece) were added to anhydrous THF (20 mL) under nitrogen. Anhydrous THF (40 mL) containing 4-bromotoluene (4.94 g, 28.9 mmol) was then dropped into the mixture. The mixture was stirred for 1.5 h at 70°C. Then, anhydrous THF (10 mL) containing diethyl carbonate (1.0 mL, 8.25 mmol) was dropped into the mixture. The mixture was stirred overnight at 50°C. Then, the reaction was quenched by the addition of saturated aqueous ammonium chloride solution, and only THF was evaporated. The mixture was extracted with dichloromethane/water, and then purified using column chromatography on silica gel with hexane/dichloromethane (1/1, v/v) as eluents to give tri-*p*-tolylmethanol (1.71 g, 68 %).

Then, the solid (1.71 g) and thionyl chloride (1.0 mL, 14 mmol) were added to chloroform (40 mL). The mixture was stirred overnight at 25°C. After the evaporation of the solvent, the residue and dichloromethane (40 mL) were added to aqueous ammonia

solution (30 wt. %, 30 mL) containing ammonium chloride (1.53 g, 28.5 mmol) at 0°C. The mixture was stirred overnight at 25°C. After the reaction, the mixture was extracted with dichloromethane/water, and then purified using column chromatography on silica gel with dichloromethane as an eluent to give a white solid. The solid was characterized as **TPMA-Me** (1.18 g, 70 %), as follows (Figure S18 and S19): ^{1}H NMR (400 MHz, chloroform-d_1, δ): 7.13 (d, J = 4.4 Hz, 6H), 7.08 (d, J = 4.4 Hz, 6H), 2.32 (s, 9H); ^{13}C NMR (400 MHz, chloroform-d_1, δ): 146.0, 136.0, 128.5, 128.0, 65.5, 20.9.

Synthesis of tris(4-chlorophenyl)methylamine (TPMA-Cl).

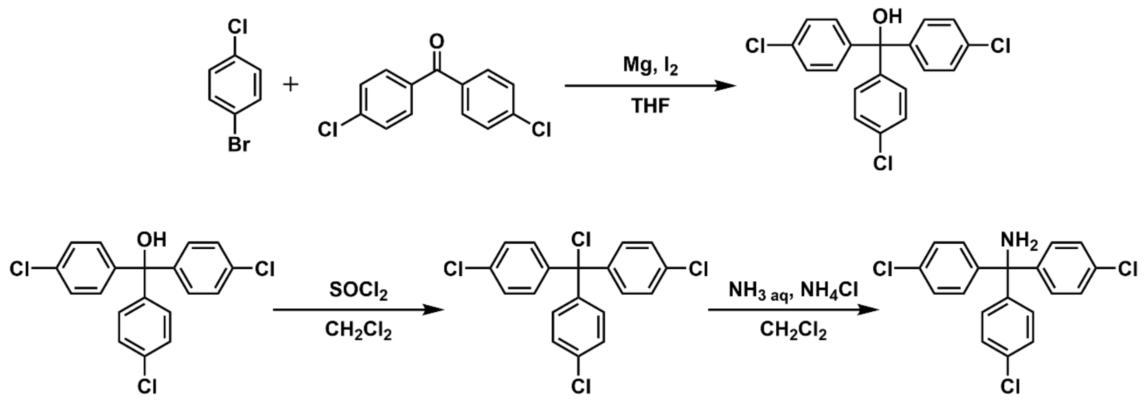

Scheme S3. Synthesis of tris(4-chlorophenyl)methylamine.

We synthesized tris(4-chlorophenyl)methylamine following the previous paper.[4] Magnesium (0.334 g, 13.7 mmol) and iodine (1 piece) were added to anhydrous THF (30 mL) under nitrogen. Anhydrous THF (20 mL) containing 4-bromochlorobenzene (2.62 g, 13.7 mmol) was then dropped into the mixture. The mixture was stirred for 1 h at 50°C. Then, anhydrous THF (20 mL) containing 4,4'-dichlorobenzophenone (2.30 g, 9.16 mmol) was dropped into the mixture. The mixture was stirred overnight at 50°C. Then, the reaction was quenched by the addition of saturated aqueous ammonium chloride solution, and only THF was evaporated. The mixture was extracted with diethyl ether/water, and then purified using column chromatography on silica gel with hexane/ethyl acetate (10/1, v/v) as eluents to give tris(4-chlorophenyl)methanol (3.20 g, 95 %).

Then, tris(4-chlorophenyl)methanol (3.20 g, 8.81 mmol) and thionyl chloride (2.0 mL, 28 mmol) were added to dichloromethane (40 mL). The mixture was stirred for 2 h at 25°C. After the evaporation of the solvent, the residue and dichloromethane (30 mL) were added to aqueous ammonia solution (30 wt. %, 30 mL) containing ammonium chloride (1.50 g, 28.0 mmol) at 0°C. The mixture was stirred overnight at 25°C. After the reaction, the mixture was extracted with dichloromethane/water, and then purified by column chromatography on silica gel with dichloromethane as eluents to give a white solid. The solid was characterized as **TPMA-Cl**[4] (2.65 g, 83 %), as follows (Figure S20 and S21): ^{1}H NMR (400 MHz, chloroform-d_1, δ): 7.27 (d, J = 8.4 Hz, 6H), 7.17 (d, J = 8.4 Hz, 6H), 2.23 (br, 2H); ^{13}C NMR (400 MHz, chloroform-d_1, δ): 146.3, 133.0, 129.3, 128.3, 65.3.

Synthesis of tris(4-bromophenyl)methylamine (TPMA-Br).

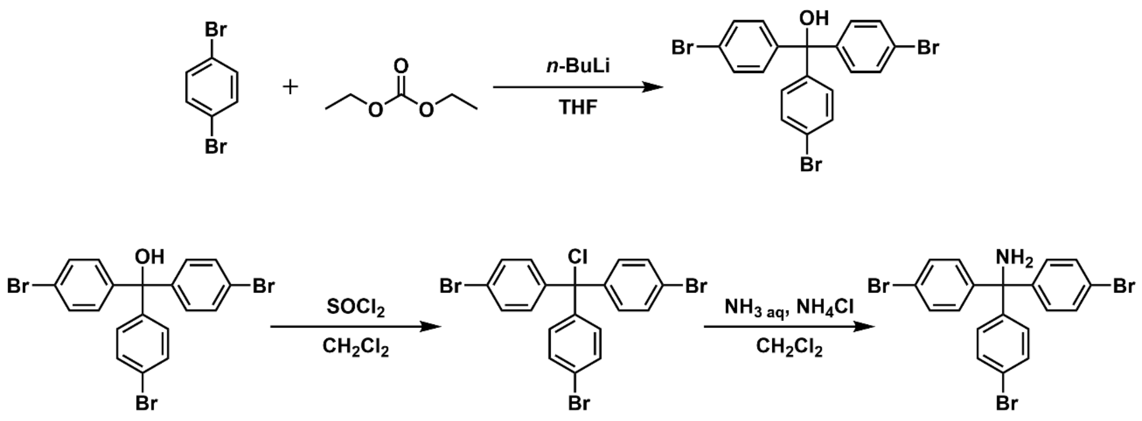

Scheme S4. Synthesis of tris(4-bromophenyl)methylamine.

We synthesized tris(4-bromophenyl)methylamine following the previous paper.[4] 1,4-Dibromobenzene (4.37 g, 18.7 mmol) was added to anhydrous THF (30 mL) under nitrogen, and then *n*-BuLi (1.6 M in hexane, 15 mL) was dropped into the mixture at -65°C. The mixture was stirred for 2 h at -65°C. Then, anhydrous THF (10 mL) containing diethyl carbonate (0.70 mL, 5.78 mmol) was dropped into the mixture. The mixture was stirred overnight at -65°C. Then, the reaction was quenched by adding saturated aqueous ammonium chloride solution, and only THF was evaporated. The mixture was extracted with ethyl acetate/water, and then purified using column chromatography on silica gel with hexane/ethyl acetate (10/1, v/v) as eluents. Then, the residue was recrystallized in hexane to give a white solid (crude, 2.43 g).

Then, the solid (2.43 g) and thionyl chloride (5.0 mL, 69 mmol) were added to chloroform (50 mL). The mixture was stirred overnight at 25°C. After the evaporation of

the solvent, the residue and dichloromethane (50 mL) were added to aqueous ammonia solution (30 wt. %, 50 mL) containing ammonium chloride (2.00 g, 37.4 mmol) at 0°C. The mixture was stirred overnight at 25°C. After the reaction, the mixture was extracted with dichloromethane/water, and then purified using column chromatography on silica gel with dichloromethane as an eluent to give a white solid. The solid was characterized as **TPMA-Br**[4] (1.78 g, 61 %), as follows (Figure S22 and S23): ^{1}H NMR (400 MHz, chloroform-d_1, δ): 7.42 (d, J = 8.8 Hz, 6H), 7.12 (d, J = 8.4 Hz, 6H), 2.21 (br, 2H); ^{13}C NMR (400 MHz, chloroform-d_1, δ): 146.7, 131.2, 129.7, 121.2, 65.4.

Synthesis of tris(4-iodophenyl)methylamine (TPMA-I).

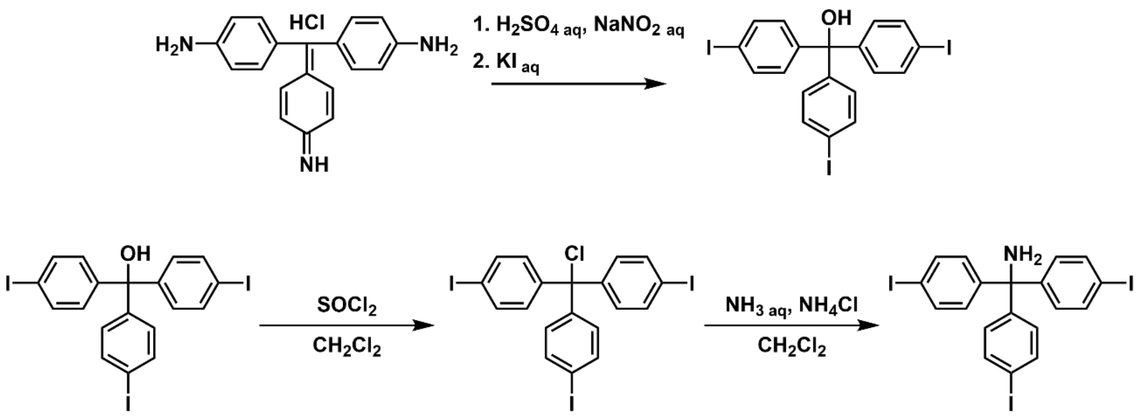

Scheme S5. Synthesis of tris(4-iodophenyl)methylamine.

We synthesized tris(4-iodophenyl)methylamine following the previous paper.[4] Sodium nitrite solution (0.794 g, 11.5 mmol in 20 mL water) was dropped into sulfonic acid (1.5 mol L^{-1}, 27 mL) containing pararosaniline hydrochloride (1.01 g, 3.30 mmol) at 0°C, and the mixture was stirred for 1 h at 0°C. Then, potassium iodide aqueous solution (5.55 g, 33.4 mmol in 20 mL water) was dropped into the mixture. The mixture was stirred for 2 h at 80°C. After the reaction, the mixture was filtered and washed with water, and the residue was purified using column chromatography on silica gel with dichloromethane as an eluent to give tris(4-iodophenyl)methanol (1.40 g, 70 %).

Then, tris(4-iodophenyl)methanol (500 mg, 0.784 mmol) and thionyl chloride (5 mL, 69 mmol) were added to chloroform (20 mL), and the mixture was stirred overnight at 25°C. After the evaporation of the solvent, the residue and dichloromethane (50 mL) were added

to aqueous ammonia solution (30 wt. %, 40 mL) containing ammonium chloride (1.07 g, 20.0 mmol) at 0°C. The mixture was stirred overnight at 25°C. After the reaction, the mixture was extracted with dichloromethane/water, and then purified using column chromatography on silica gel with dichloromethane as an eluent to give a white solid. The solid was characterized as **TPMA-I**[4] (366 mg, 73 %), as follows (Figure S24 and S25): ^{1}H NMR (400 MHz, chloroform-d_1, δ): 7.61 (d, J = 8.0 Hz, 6H), 6.98 (d, J = 8.0 Hz, 6H), 2.18 (br, 2H); ^{13}C NMR (400 MHz, chloroform-d_1, δ): 147.4, 137.4, 130.0, 92.8.

Preparation of 4,4',4'',4'''-(adamantane-1,3,5,7-tetrayl)tetrabenzenesulfonic acid (AdPS).

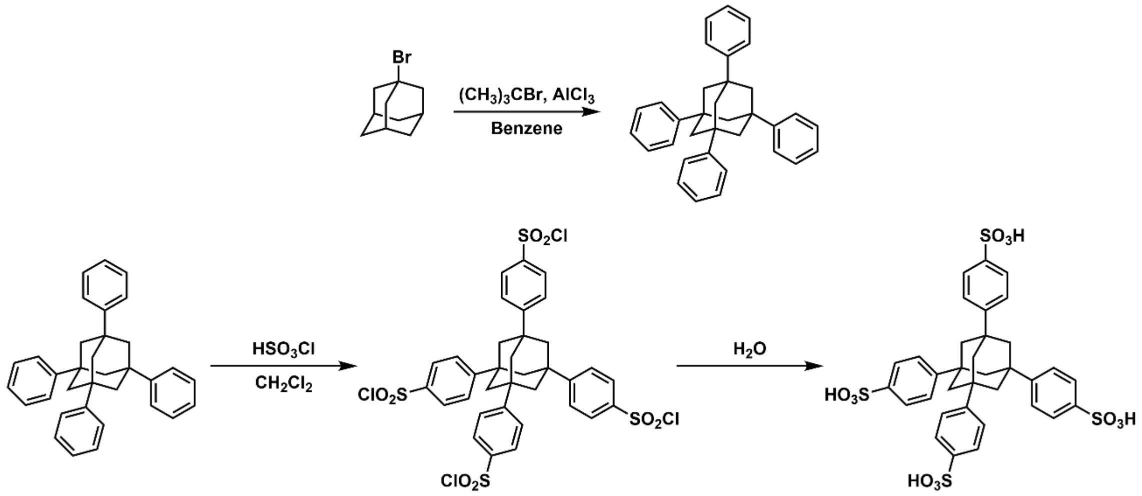

Scheme S6. Synthesis of 4,4',4'',4'''-(adamantane-1,3,5,7-tetrayl)tetrabenzenesulfonic acid.

We synthesized 4,4',4'',4'''-(adamantane-1,3,5,7-tetrayl)tetrabenzenesulfonic acid following the previous paper.[5] 1-Bromo adamantane (6.03 g, 28.0 mmol) and aluminum chloride anhydrous (1.65 g, 12.4 mmol) were suspended in anhydrous benzene (100 mL) under nitrogen, and then *t*-Butylbromide (10 mL, 89.0 mmol) was dropped into the mixture. The mixture was refluxed overnight. The reaction mixture was cooled to 25°C and the formed precipitate was filtered off and washed with chloroform and water to give a tetraphenyladamantane (12.0 g, 97 %).

Then, chlorosulfonic acid (2.3 mL, 34.5 mmol) was dropped into the suspension of tetraphenyladamantane (1.50 g, 3.40 mmol) and dichloromethane (40 mL) at 0°C. The mixture was heated to 35°C for 80 min. The reaction mixture was cooled to 25°C and centrifugated to separate a brown oil from yellow dichloromethane solution. The yellow

supernatant was decanted and evaporated the solvent at 25°C. After evaporation, cooled hydrochloric acid (1M, 100 mL) was added and a white solid was precipitated by sonication. The precipitate was filtered off and dried in vacuo at 25°C for 24 h, and then purified using column chromatography on silica gel with dichloromethane as an eluent to give a white solid (0.85 g, 30%).

Then 4,4',4'',4'''-(adamantane-1,3,5,7-tetrayl)tetrabenzenesulfonyl chloride (0.85 g, 1.02 mmol) was suspended in water (150 mL) and refluxed for 48 h to give a clear solution. The solution was evaporated and then azeotropic removal of hydrochloric acid was performed with dioxane to give an ivory solid. The solid was characterized as **AdPS**[5b] (0.76 g, 99%), as follows (Figure S26 and S27): ^{1}H NMR (400 MHz, methanol-d_4, δ): 7.83 (d, J = 8.4 Hz, 8H), 7.66 (d, J = 8.4 Hz, 8H), 2.22 (s, 12H); ^{13}C NMR (400 MHz, D_2O, δ): 153.8, 140.8, 126.6, 126.2, 46.4, 40.0.

Preparation of the organic salts composed of AdPS and TPMA.

TPMA (5 eq) was dissolved in methanol. In the different vessels, **AdPS** (1 eq) was dissolved in methanol. Then, these two different solutions were mixed. After the evaporation of the solvent and washing the residue with diethyl ether, the organic salts were obtained as a white powder.

Preparation of the organic salts composed of AdPS and TPMA-X.

TPMA-X (5 eq) was dissolved in methanol. In the different vessels, **AdPS** (1 eq) was dissolved in methanol. Then, these two different solutions were mixed. After the evaporation of the solvent and washing the residue with diethyl ether, the organic salts were obtained as a powder.

Preparation of the single-crystal of AdPS/TPMA.

The organic salt (2.0 mg) was recrystallized in dimethyl sulfoxide (200 μL) by slow evaporation of the solvent at 70°C. The single crystals of **AdPS/TPMA** were obtained as a colorless block crystal.

Preparation of the single-crystal of AdPS/TPMA-F.

The single crystals of **AdPS/TPMA-F** (***dia***-, ***lon***-, and ***uni***-topology) were obtained by the following methods.

dia-topology: The organic salt (2.0 mg) was recrystallized with pentafluorobenzonitrile (150 μL) as a template molecule in methanol (200 μL) by slow evaporation of the solvent at 25°C to obtain the single crystals as a colorless block crystal.

lon-topology: The organic salt (2.0 mg) was recrystallized with (+)-3-carene (150 μL) as a template molecule in methanol (200 μL) by slow evaporation of the solvent at 25°C to obtain the single crystals as a colorless block crystal.

uni-topology with left-handed helical structure: The organic salt (2.0 mg) was recrystallized with (-)-carvone (150 μL) as a template molecule in methanol (200 μL) by slow evaporation of the solvent at 25°C to obtain the single crystals as a colorless block crystal.

uni-topology with right-handed helical structure: The organic salt (2.0 mg) was recrystallized with (+)-carvone (150 μL) as a template molecule in methanol (200 μL) by slow evaporation of the solvent at 25°C to obtain the single crystals as a colorless block crystal.

Preparation of the single-crystal of AdPS/TPMA-Me.

The single crystals of **AdPS/TPMA-Me** (***dia***-, ***lon***-, and ***sod***-topology) were obtained by the following methods.

dia-topology: The organic salt (2.0 mg) was recrystallized with (-)-β-pinene (150 μL) as template molecule in methanol (200 μL) by slow evaporation of the solvent at 25°C to obtain the single crystals as a colorless block crystal.

lon-topology: The organic salt (2.0 mg) was recrystallized with (+)-3-carene (150 μL) as template molecule in methanol (200 μL) by slow evaporation of the solvent at 25°C to obtain the single crystals as a colorless block crystal.

sod-topology: The organic salt (2.0 mg) was recrystallized with benzonitrile (150 μL) as template molecule in methanol (200 μL) by slow evaporation of the solvent at 25°C to obtain the single crystals as a colorless block crystal.

Preparation of the single-crystal of AdPS/TPMA-Cl.

The organic salt (2.0 mg) was recrystallized with mesitylene (150 μL) as template molecule in methanol (200 μL) by slow evaporation of the solvent at 25°C to obtain the single crystals of **AdPS/TPMA-Cl** as a colorless block crystal.

Preparation of the single-crystal of AdPS/TPMA-Br.

The organic salt (2.0 mg) was recrystallized with mesitylene (150 μL) as template molecule in methanol (200 μL) by slow evaporation of the solvent at 25°C to obtain the single crystals of **AdPS/TPMA-Br** as a colorless block crystal.

Preparation of the single-crystal of AdPS/TPMA-I.

The organic salt (2.0 mg) was recrystallized with nitrobenzene (150 μL) as template molecule in *N,N*-dimethylacetamide (200 μL) by slow evaporation of the solvent at 70°C to obtain the single crystals of **AdPS/TPMA-I** as a colorless block crystal.

Element analyses of AdPS/TPMA-Me with dia, lon, and sod-topologies.

AdPS/TPMA-Me with ***dia***-topology: Elemental Analysis calcd. [($C_{34}H_{32}O_{12}S_4$) · 4($C_{22}H_{23}N$) · 6(H_2O)] %C 70.63, %H 6.61, %N 2.70, %S 6.18 found %C 70.45, %H 6.23, %N 2.70, %S 6.45.

AdPS/TPMA-Me with ***lon***-topology: Elemental Analysis calcd. [($C_{34}H_{32}O_{12}S_4$) · 4($C_{22}H_{23}N$) · 8(H_2O)] %C 69.42, %H 6.69, %N 2.65, %S 6.08 found %C 69.42, %H 6.24, %N 2.67, %S 6.46.

AdPS/TPMA-Me with ***sod***-topology: Elemental Analysis calcd. [($C_{34}H_{32}O_{12}S_4$) · 4($C_{22}H_{23}N$) · 6(H_2O)] %C 70.63, %H 6.61, %N 2.70, %S 6.18 found %C 70.63, %H 6.36, %N 2.76, %S 6.20.

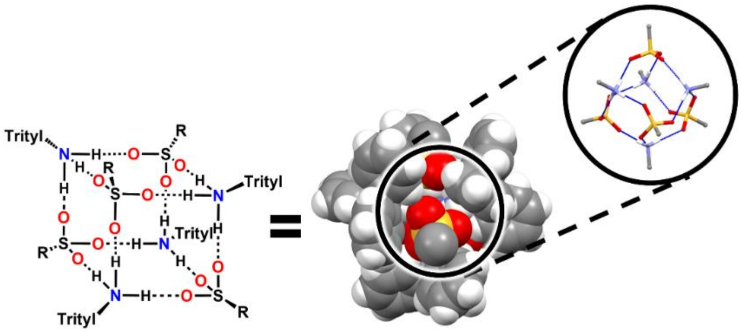

Figure S1. Corey-Pauling-Koltun (**CPK**) molecular model[6] of supramolecular cluster with four sulfonic acids and four **TPMA** in **POS**s.

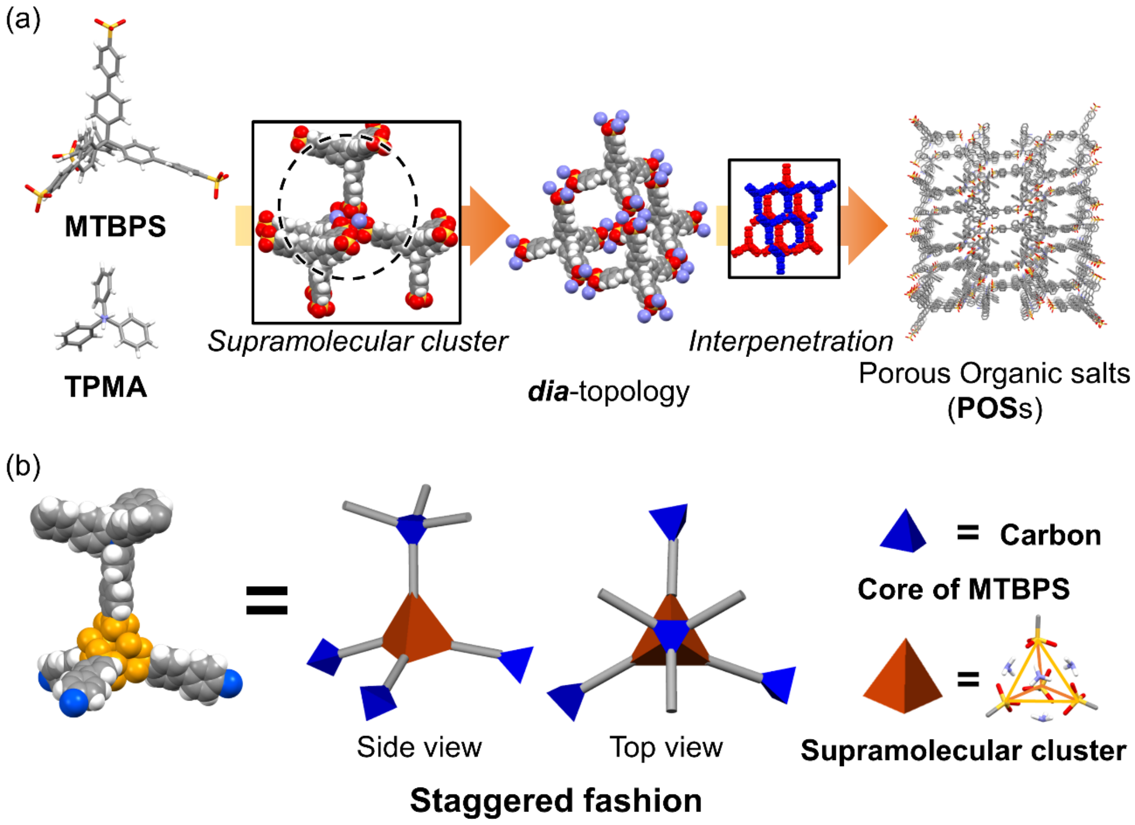

Figure S2. (a) Schematic representation of the construction and the resultant porous structure with ***dia***-topology of the salt composed of 4',4''',4''''',4'''''''-methanetetrayltetrakis (([1,1'-biphenyl]-4-sulfonic acid)) (**MTBPS**) and **TPMA**. (b) The conformation with staggered fashion between carbon core of **MTBPS** and supramolecular cluster.

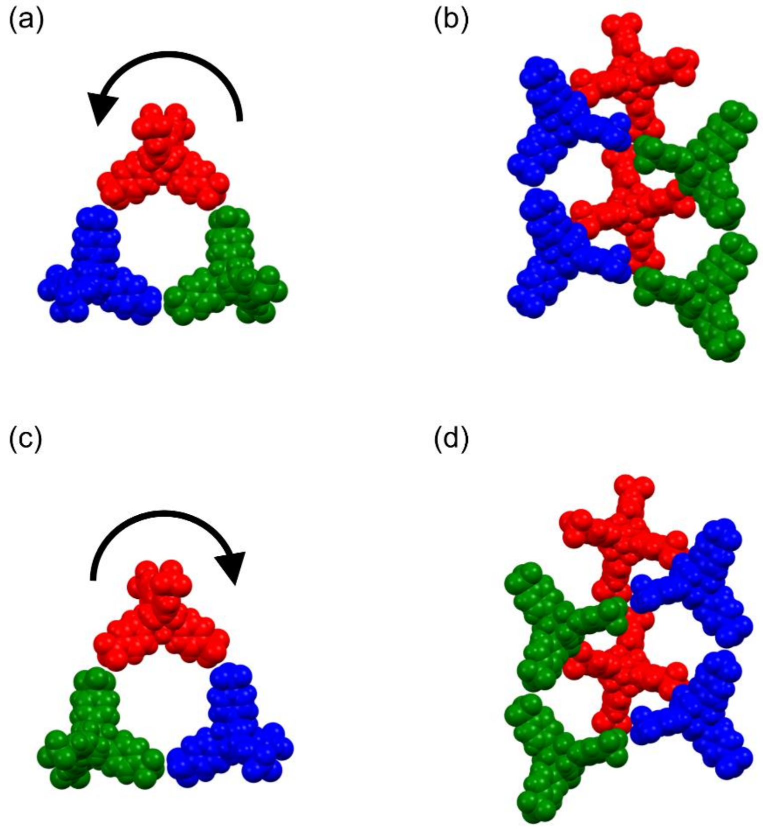

Figure S3. CPK molecular model of helical structure in **AdPS/TPMA-F** with ***uni***-topology: top view (a) and side view (b) of left-handed helical structure, and top view (c) and side view (d) of right-handed helical structure.

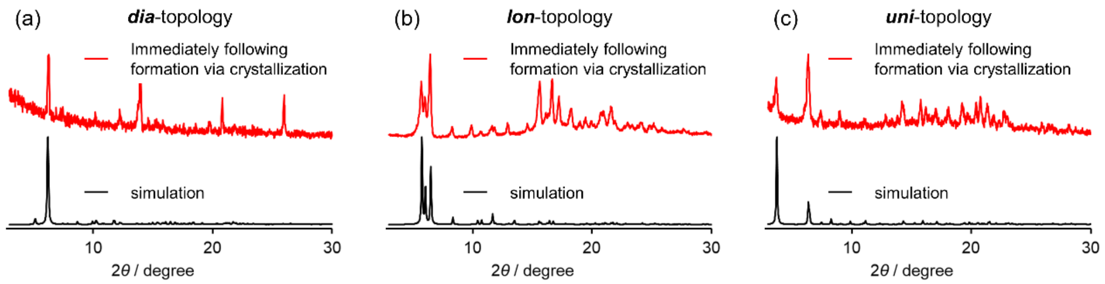

Figure S4. PXRD patterns of ***dia***-topology (a), ***lon***-topology (b), and ***uni***-topology (c) of **AdPS/TPMA-F**: simulation (black), immediately following formation via crystallization (red).

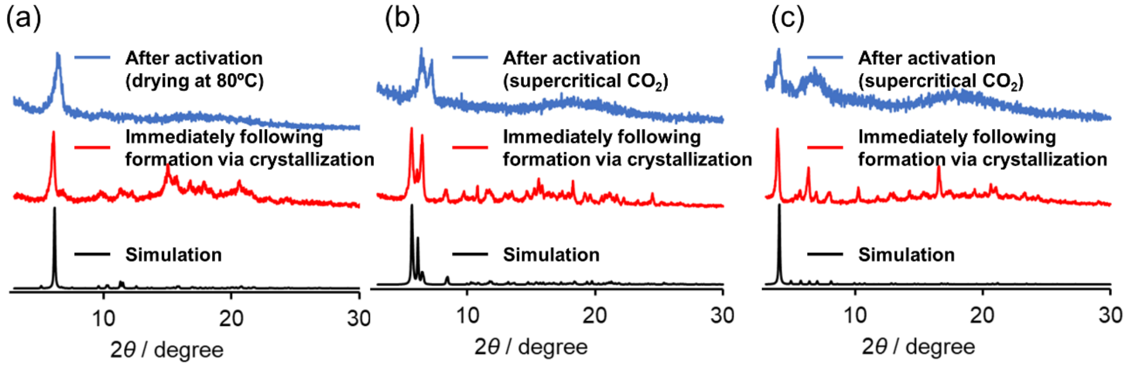

Figure S5. PXRD patterns of ***dia***-topology (a), ***lon***-topology (b), and ***sod***-topology (c) of **AdPS/TPMA-Me**: simulation (black), immediately following formation via crystallization (red), after activation (blue). ***dia***-topology was activated by drying at 80ºC, and ***lon***-topology and ***sod***-topology were activated by supercritical CO_2 fluid.

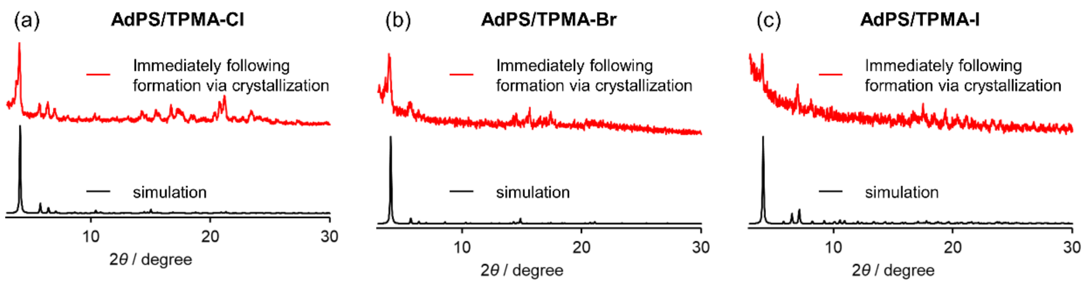

Figure S6. PXRD patterns of ***sod***-topology of **AdPS/TPMA-Cl** (a), **AdPS/TPMA-Br** (b), and **AdPS/TPMA-I** (c): simulation (black), immediately following formation via crystallization (red).

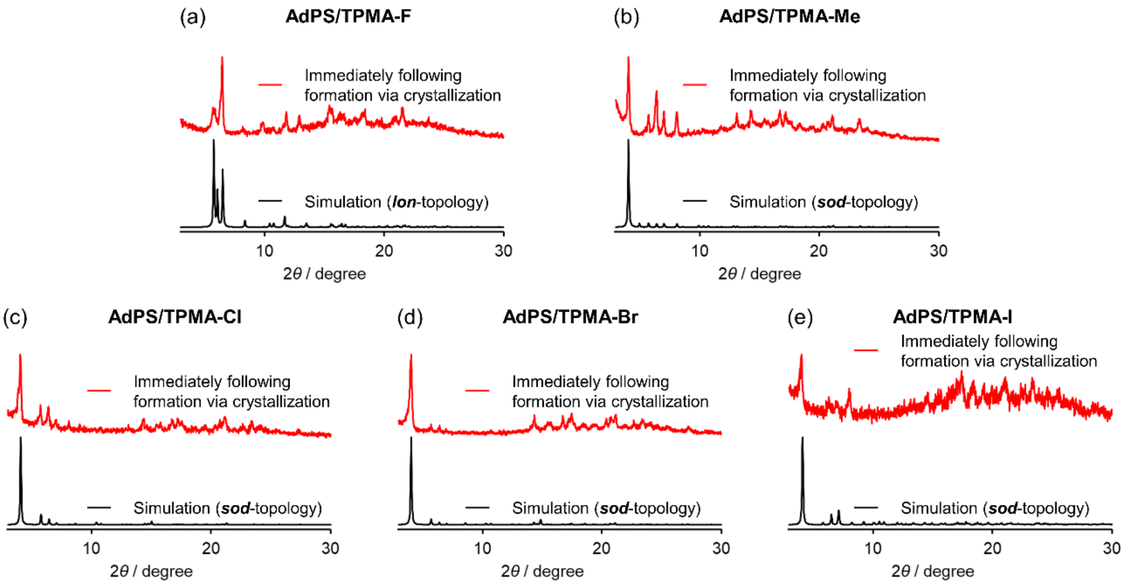

Figure S7. PXRD patterns of **AdPS/TPMA-F** (a), **AdPS/TPMA-Me** (b), **AdPS/TPMA-Cl** (c), **AdPS/TPMA-Br** (d), and **AdPS/TPMA-I** (e) which were prepared by the same condition (template: 1-methylnaphthalen, solvent: methanol, temperature: 25°C): simulation (black), immediately following formation via crystallization (red).

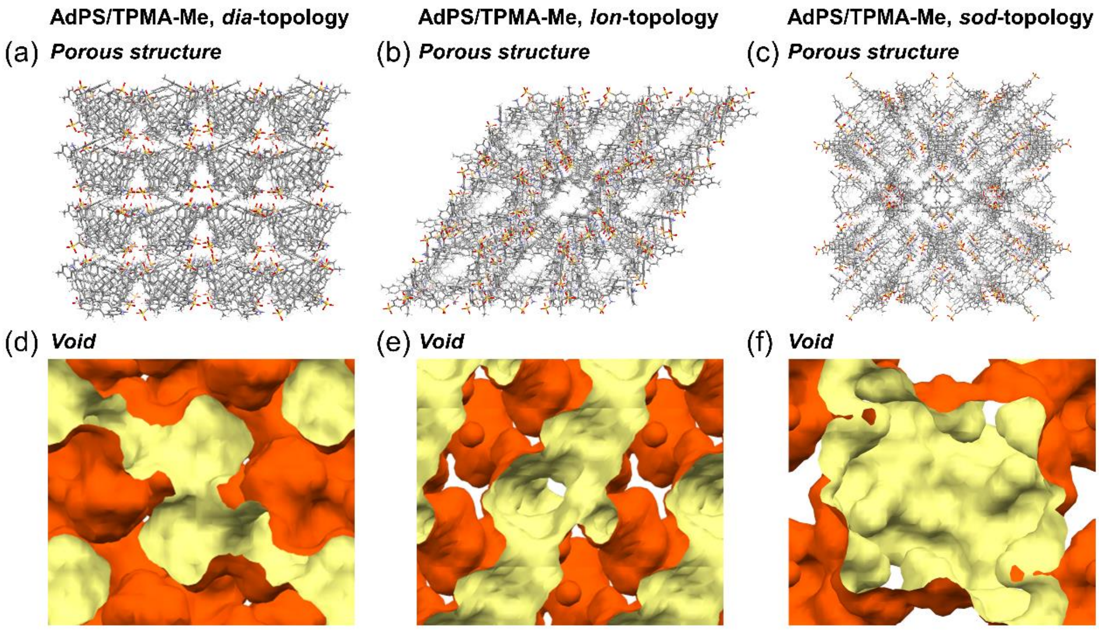

Figure S8. Schematic representations of porous structures and void structures of **AdPS/TPMA-Me** with (a), (d) ***dia***-topology, (b), (e) ***lon***-topology, and (c), (f) ***sod***-topology.

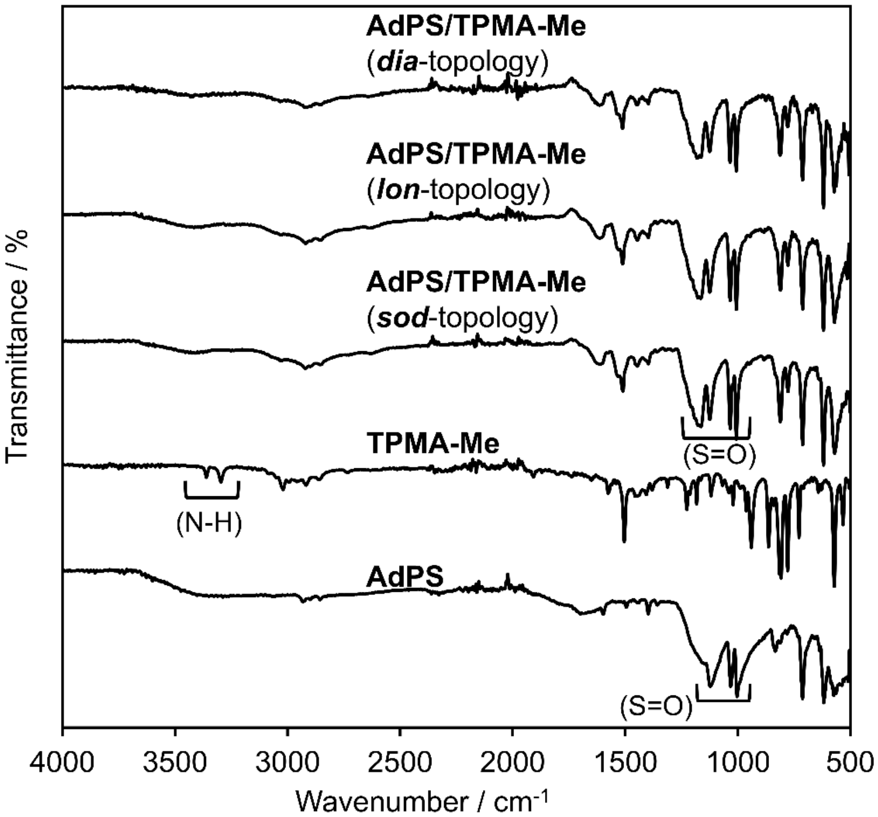

Figure S9. FT-IR spectra of **AdPS/TPMA-Me** with ***dia***-, ***lon***-, and ***sod***-topologies after activation, **TPMA-Me**, and **AdPS**.

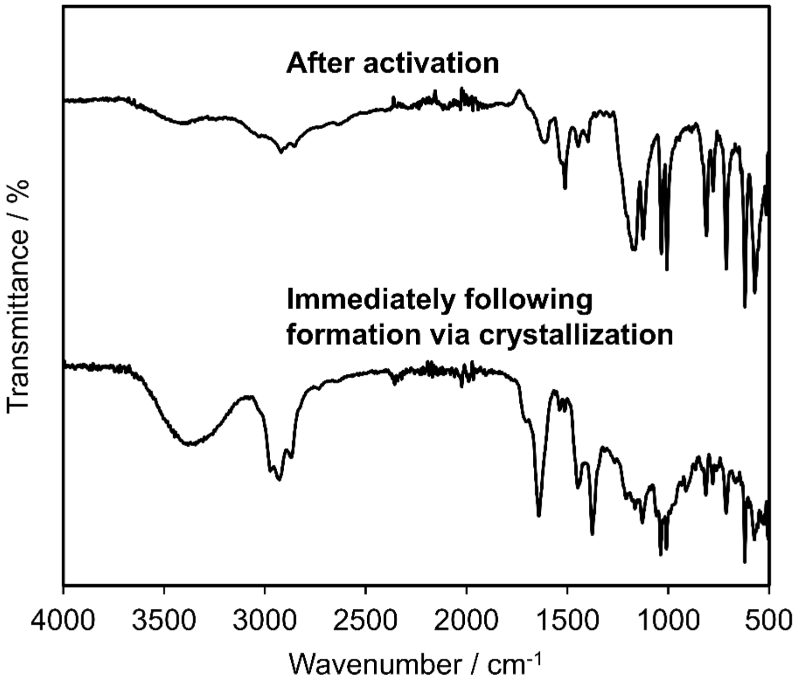

Figure S10. FT-IR spectra of **AdPS/TPMA-Me** with ***lon***-topology immediately following formation via crystallization and after activation.

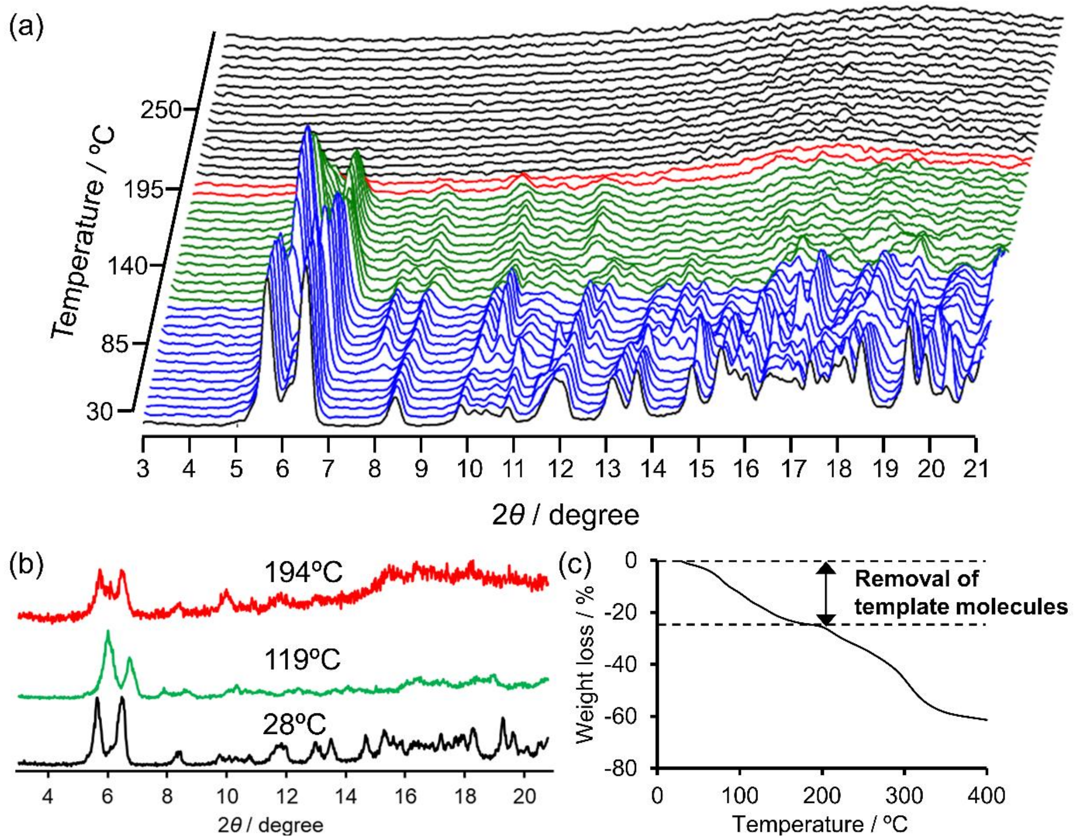

Figure S11. (a) Variable-temperature (**VT**) **PXRD** patterns of **AdPS/TPMA-Me** with ***lon***-topology. (b) Selected patterns representative of the observed phases from **VTPXRD** patterns. (c) Thermogravimetric analysis (**TGA**) data of **AdPS/TPMA-Me** with ***lon***-topology.

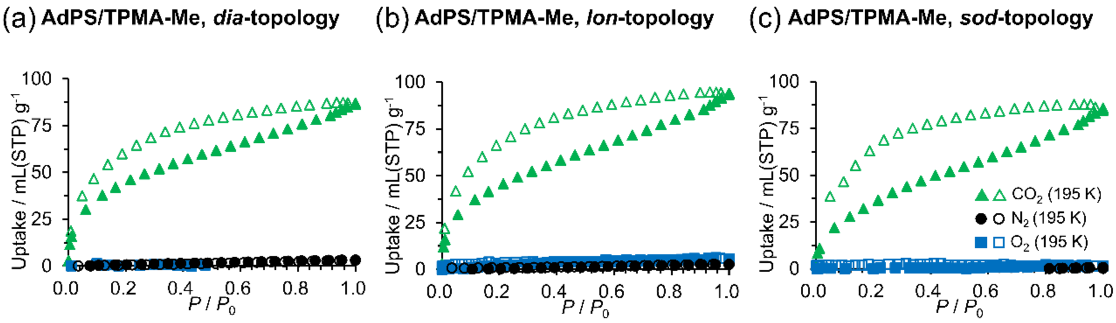

Figure S12. Gas adsorption isotherms of ***dia***-topology (a), ***lon***-topology (b), and ***sod***-topology (c) of **AdPS/TPMA-Me**: CO_2, N_2, and O_2 at 195 K. Filled symbols: adsorption process, open symbols: desorption process. P denotes the pressure at adsorption and P_0 denotes the atmospheric pressure.

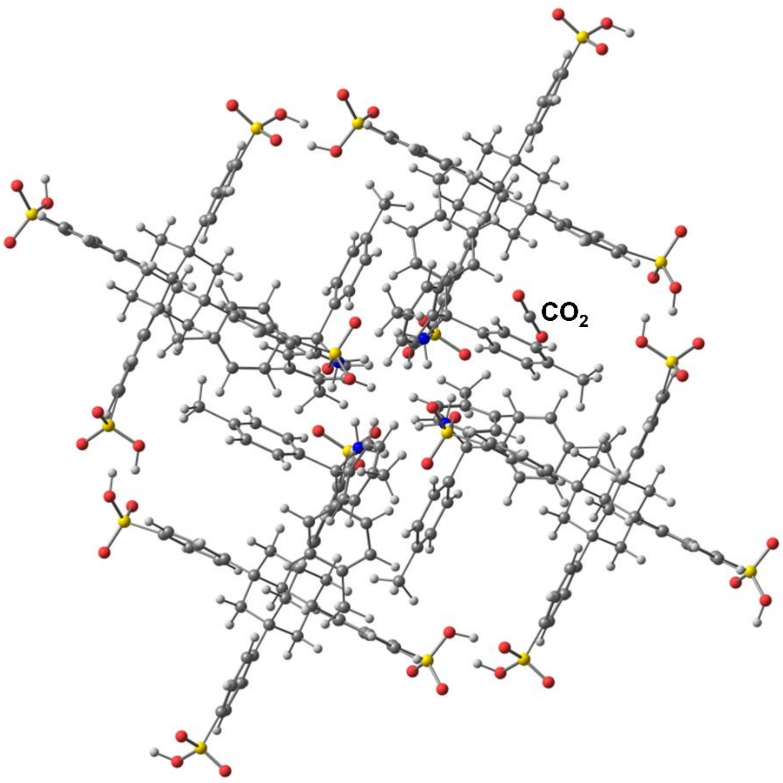

Figure S13. Optimized structures of the adsorption of CO_2 on the supramolecular cluster of **AdPS/TPMA-Me** with ***dia***-topology.

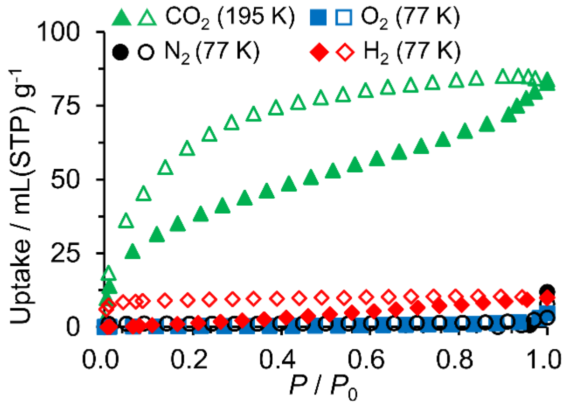

Figure S14. Gas adsorption isotherms of **AdPS/TPMA-F** with ***lon***-topology: CO_2 (195 K), N_2 (77 K), O_2 (77 K), H_2 (77 K). Filled symbols: adsorption process, open symbols: desorption process. P denotes the pressure at adsorption and P_0 denotes the atmospheric pressure.

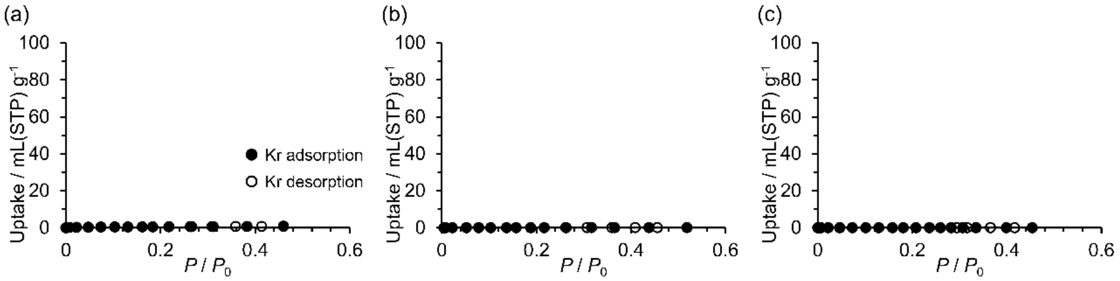

Figure S15. Krypton gas adsorption isotherms of **AdPS/TPMA-Me** with ***dia***-topology (a), ***lon***-topology (b), and ***sod***-topology (c) at 77 K. Filled symbols: adsorption process, open symbols: desorption process. *P* denotes the pressure at adsorption and P_0 denotes the saturated vapor pressure of Kr at 77 K.

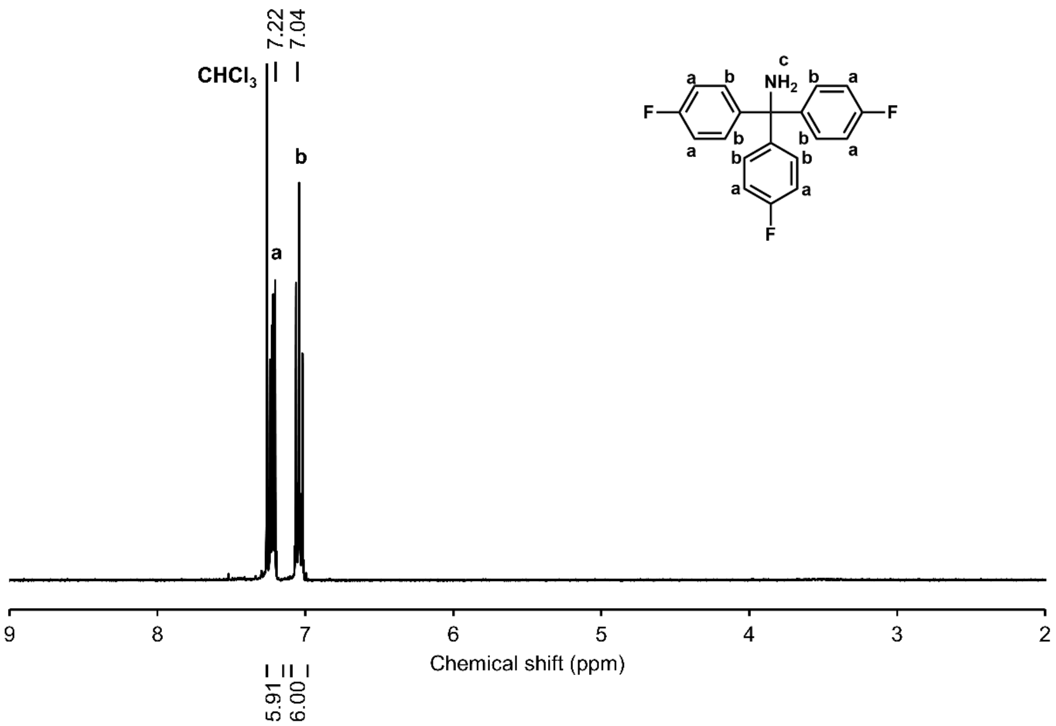

Figure S16. 400 MHz ^{1}H NMR spectrum (chloroform-d_1) of tris(4-fluorophenyl)methylamine (**TPMA-F**): ^{1}H NMR (400 MHz, chloroform-d_1, δ): 7.22 (dd, J = 8.8 Hz, 6H), 7.04 (t, J = 8.8 Hz, 6H).

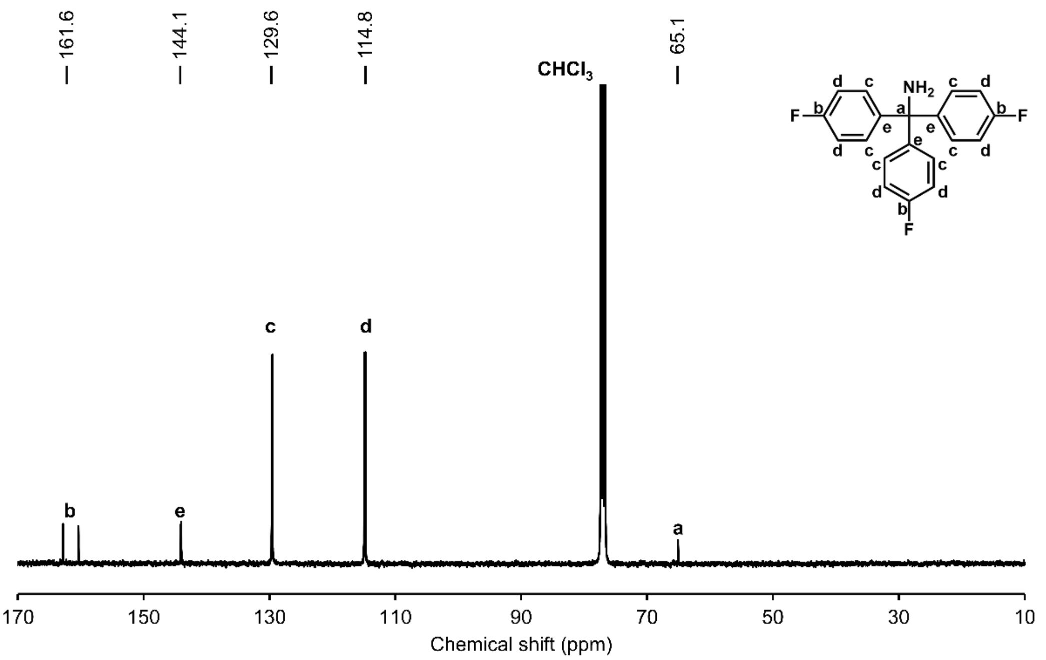

Figure S17. 400 MHz ^{13}C NMR spectrum (chloroform-d_1) of tris(4-fluorophenyl)methylamine (**TPMA-F**): ^{13}C NMR (400 MHz, chloroform-d_1, δ): 161.6, 144.1, 129.6, 114.8, 65.1.

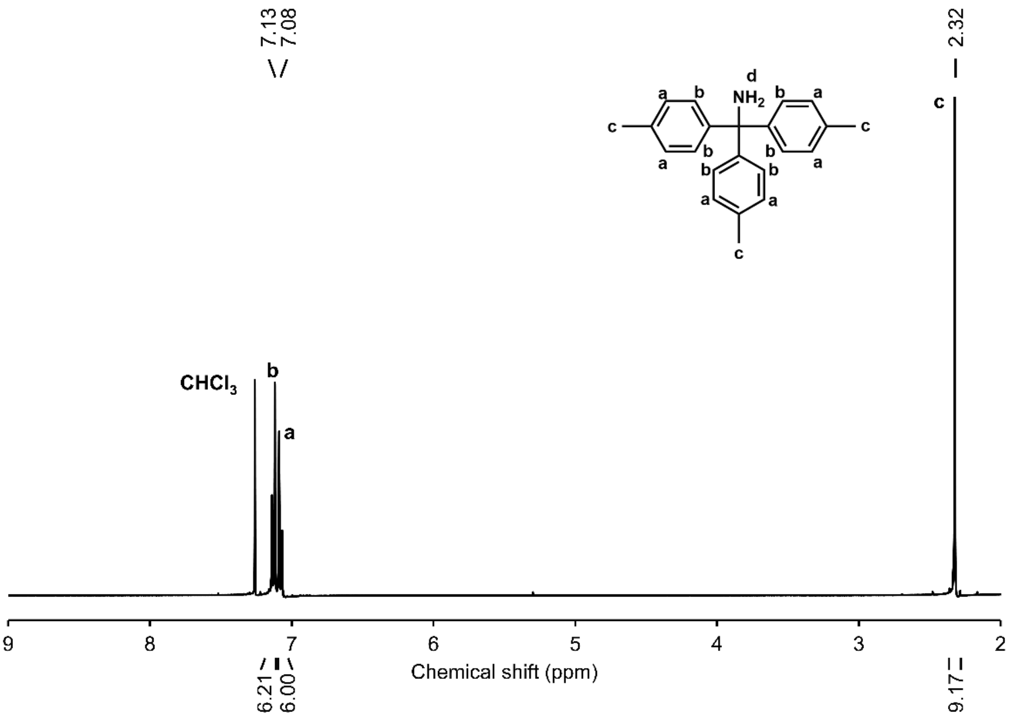

Figure S18. 400 MHz ^{1}H NMR spectrum (chloroform-d_1) of tri-*p*-tolylmethanamine (**TPMA-Me**): ^{1}H NMR (400 MHz, chloroform-d_1, δ): 7.13 (d, J = 4.4 Hz, 6H), 7.08 (d, J = 4.4 Hz, 6H), 2.32 (s, 9H).

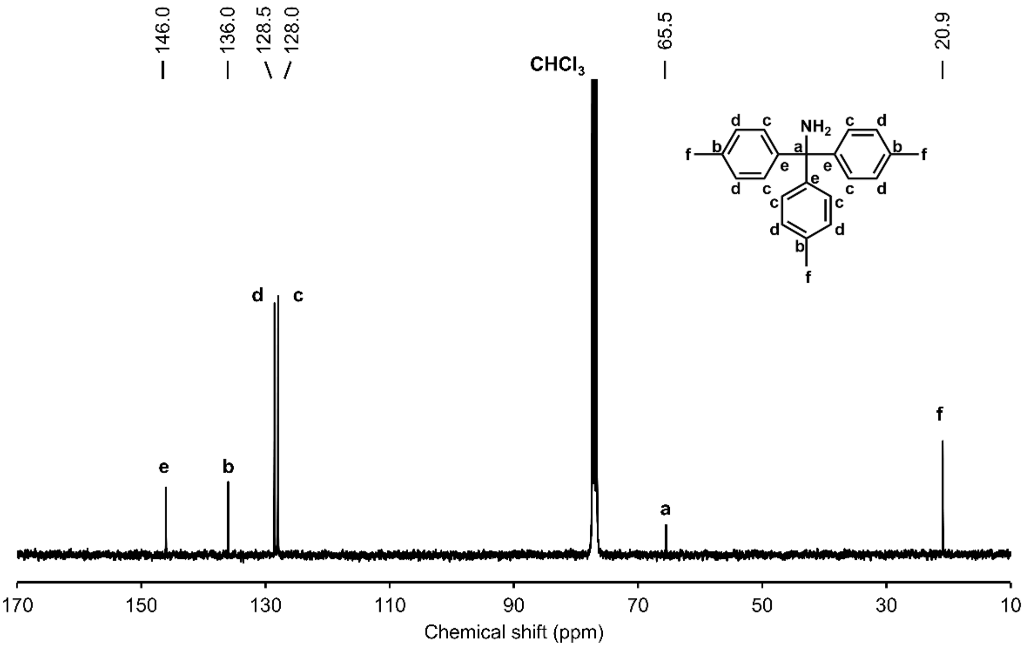

Figure S19. 400 MHz ^{13}C NMR spectrum (chloroform-d_1) of tri-*p*-tolylmethanamine (**TPMA-Me**): ^{13}C NMR (400 MHz, chloroform-d_1, δ): 146.0, 136.0, 128.5, 128.0, 65.5, 20.9.

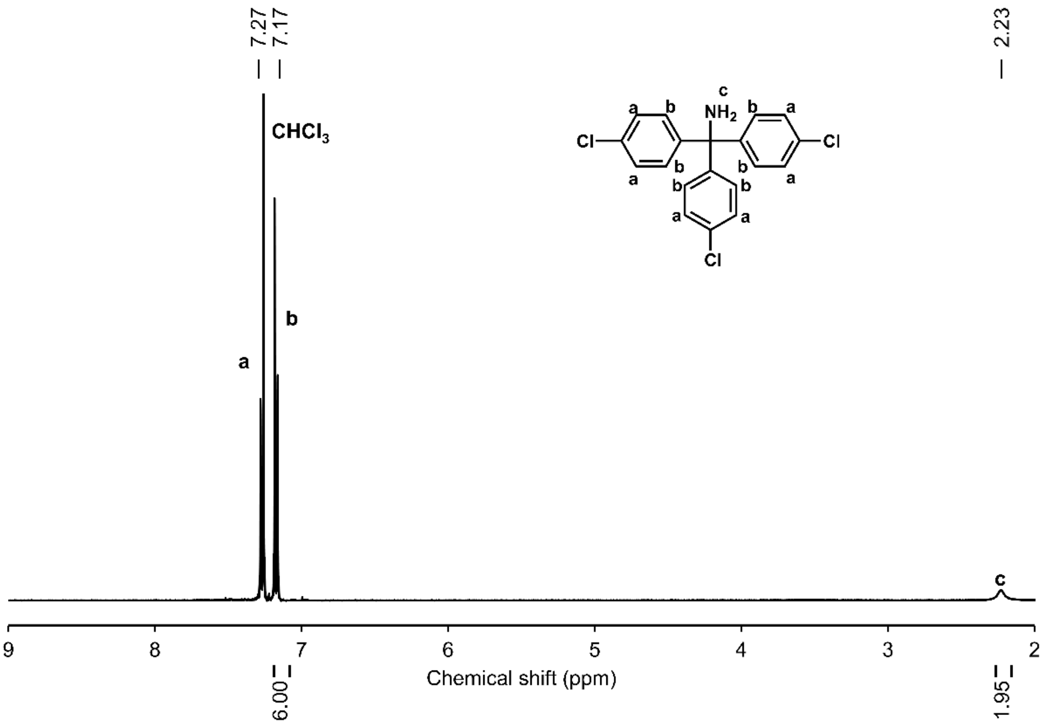

Figure S20. 400 MHz ^{1}H NMR spectrum (chloroform-*d*1) of tris(4-chlorophenyl)methylamine (**TPMA-Cl**): ^{1}H NMR (400 MHz, chloroform-*d*1, *δ*): 7.27 (d, *J* = 8.4 Hz, 6H), 7.17 (d, *J* = 8.4 Hz, 6H), 2.23 (br, 2H).

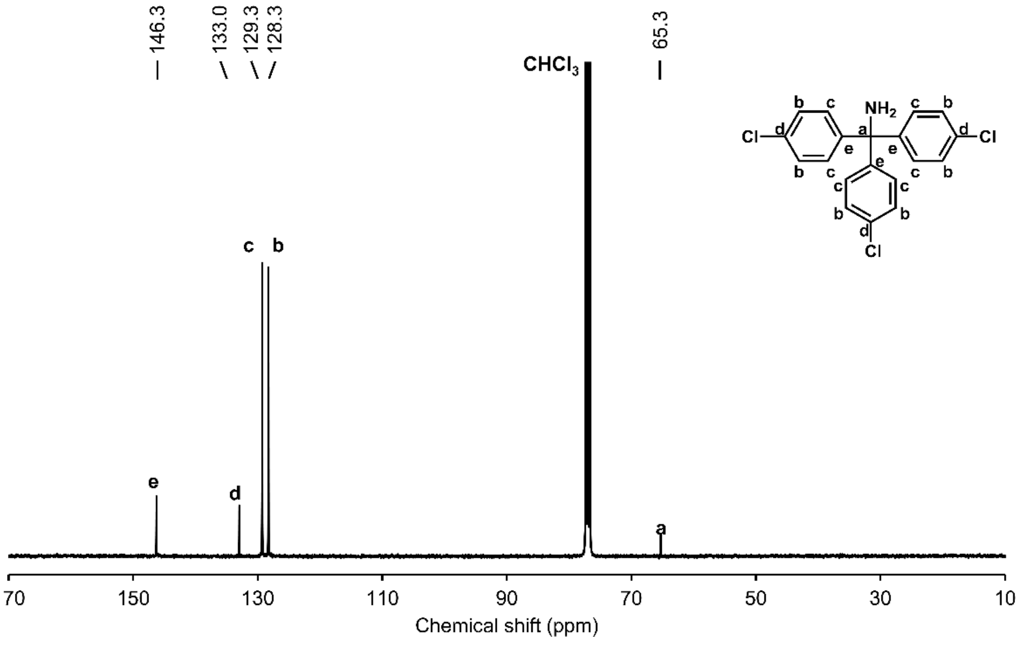

Figure S21. 400 MHz ^{13}C NMR spectrum (chloroform-*d*1) of tris(4-chlorophenyl)methylamine (**TPMA-Cl**): ^{13}C NMR (400 MHz, chloroform-*d*1, *δ*): 146.3, 133.0, 129.3, 128.3, 65.3.

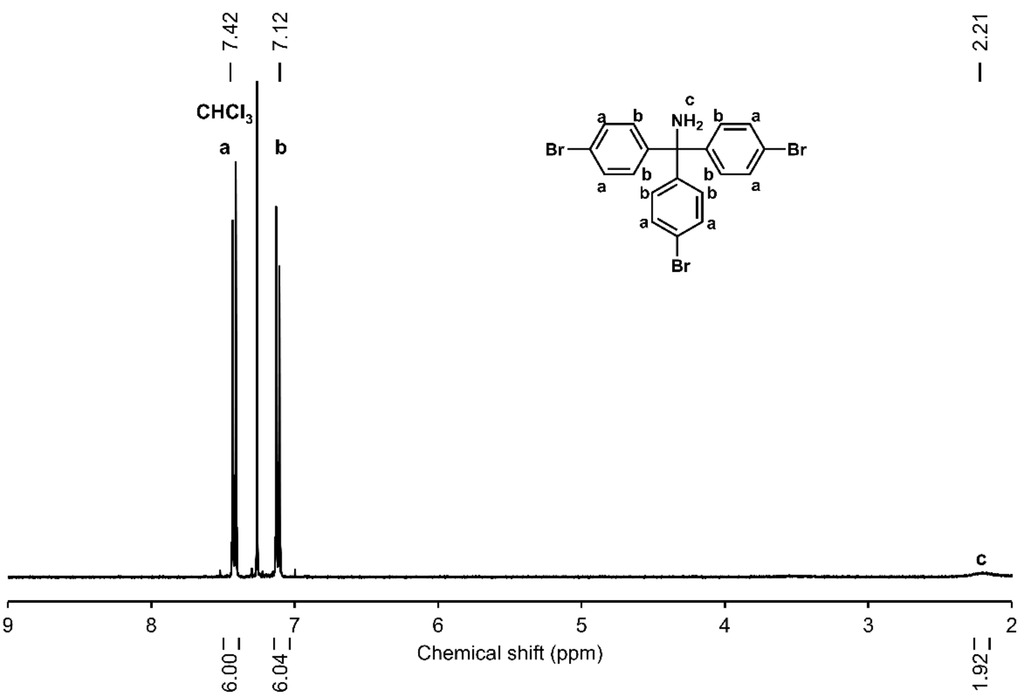

Figure S22. 400 MHz ^{1}H NMR spectrum (chloroform-d_1) of tris(4-bromophenyl)methylamine (**TPMA-Br**): ^{1}H NMR (400 MHz, chloroform-d_1, δ): 7.42 (d, J = 8.8 Hz, 6H), 7.12 (d, J = 8.4 Hz, 6H), 2.21 (br, 2H).

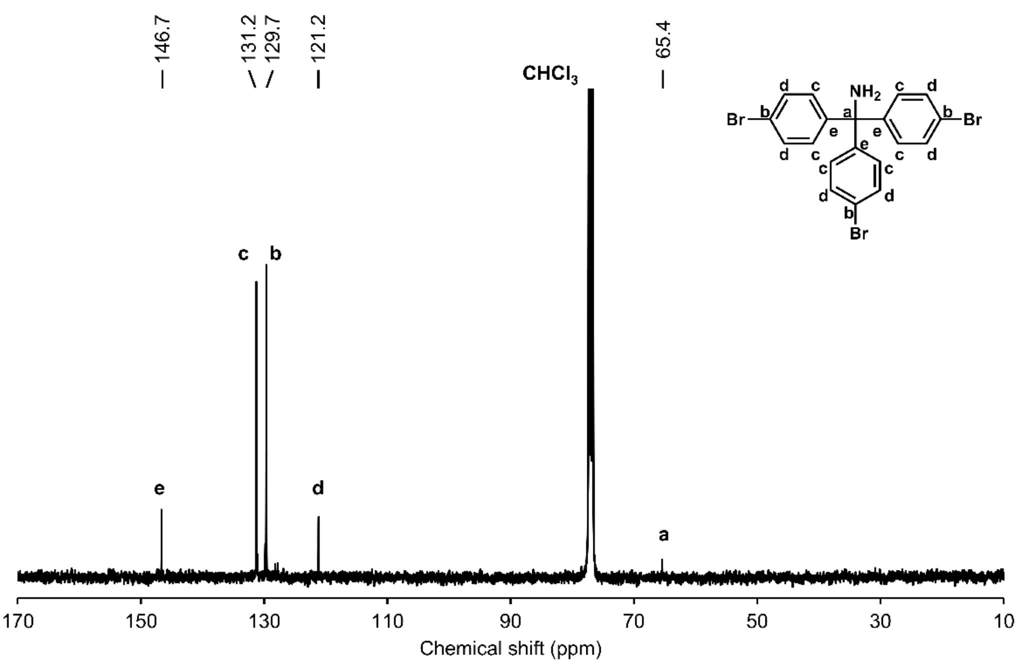

Figure S23. 400 MHz ^{13}C NMR spectrum (chloroform-d_1) of tris(4-bromophenyl)methylamine (**TPMA-Br**): ^{13}C NMR (400 MHz, chloroform-d_1, δ): 146.7, 131.2, 129.7, 121.2, 65.4.

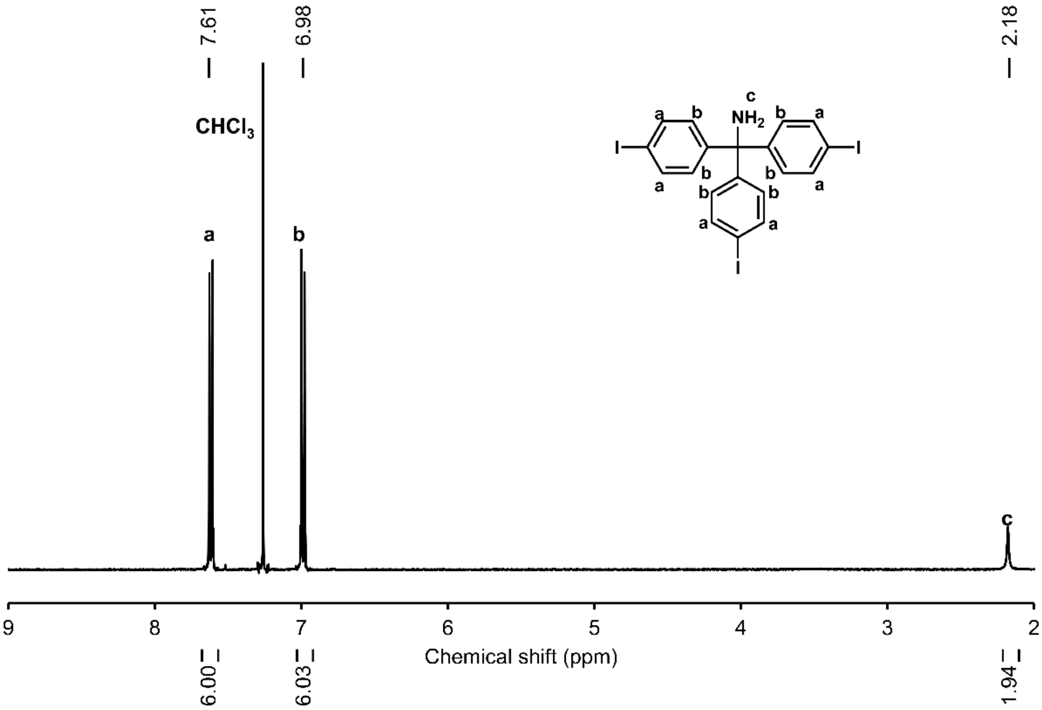

Figure S24. 400 MHz ^{1}H NMR spectrum (chloroform-*d*1) of tris(4-iodophenyl)methylamine (**TPMA-I**): ^{1}H NMR (400 MHz, chloroform-*d*1, *δ*): 7.61 (d, *J* = 8.0 Hz, 6H), 6.98 (d, *J* = 8.0 Hz, 6H), 2.18 (br, 2H).

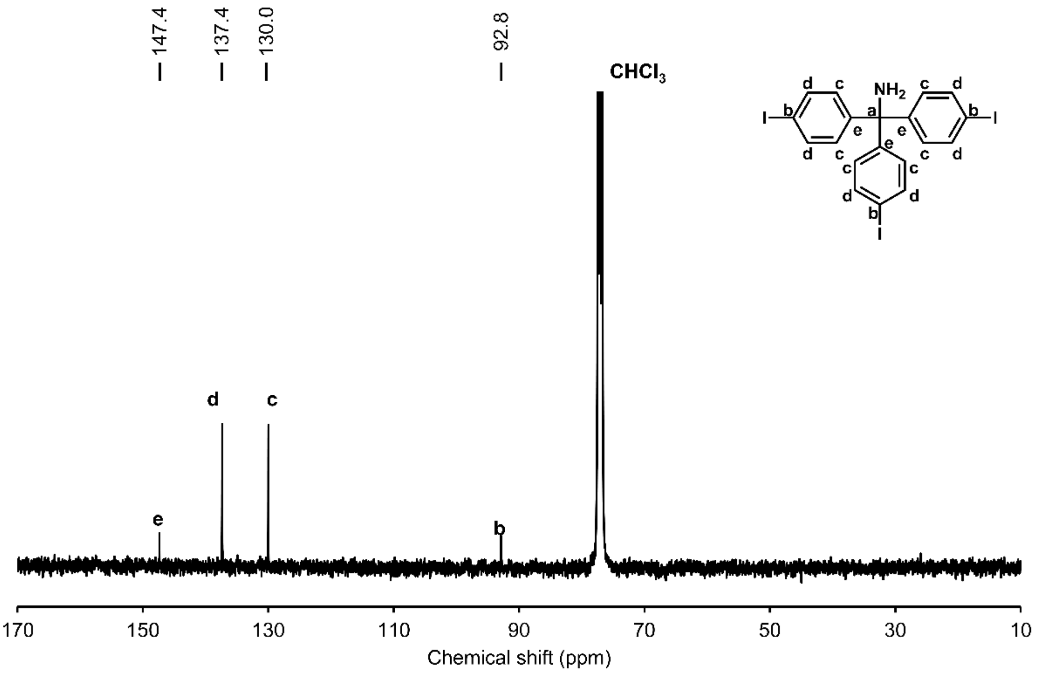

Figure S25. 400 MHz ^{13}C NMR spectrum (chloroform-*d*1) of tris(4-iodophenyl)methylamine (**TPMA-I**): ^{13}C NMR (400 MHz, chloroform-*d*1, *δ*): 147.4, 137.4, 130.0, 92.8.

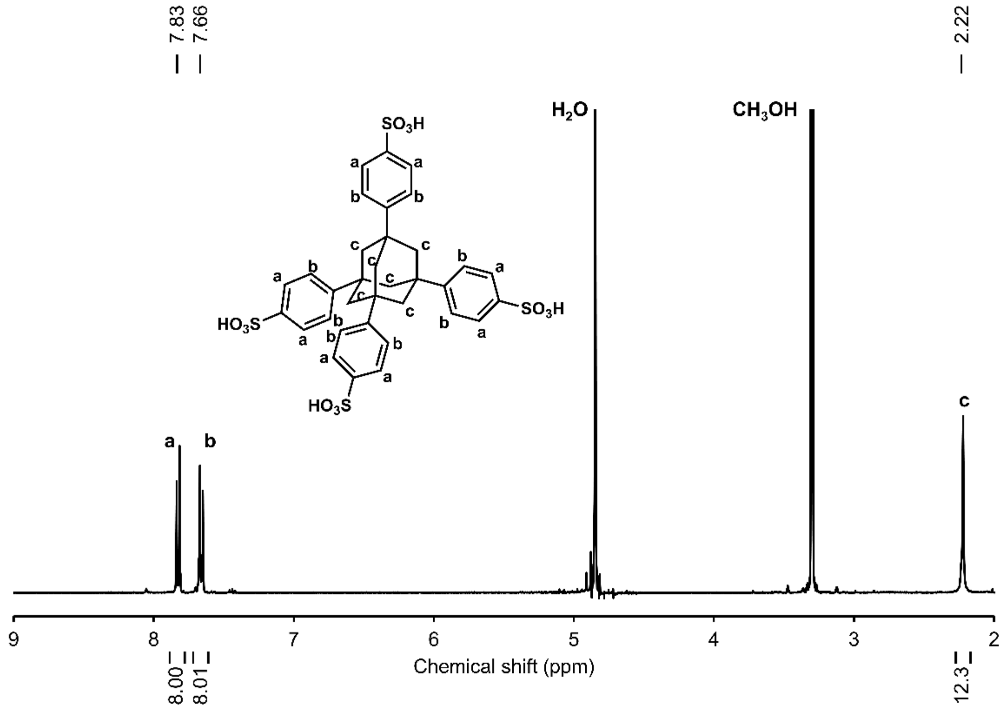

Figure S26. 400 MHz ^{1}H NMR spectrum (methanol-d_4) of 4,4',4'',4'''-(adamantane-1,3,5,7-tetrayl)tetrabenzenesulfonic acid (**AdPS**): ^{1}H NMR (400 MHz, methanol-d_4, δ): 7.83 (d, J = 8.4 Hz, 8H), 7.66 (d, J = 8.4 Hz, 8H), 2.22 (s, 12H).

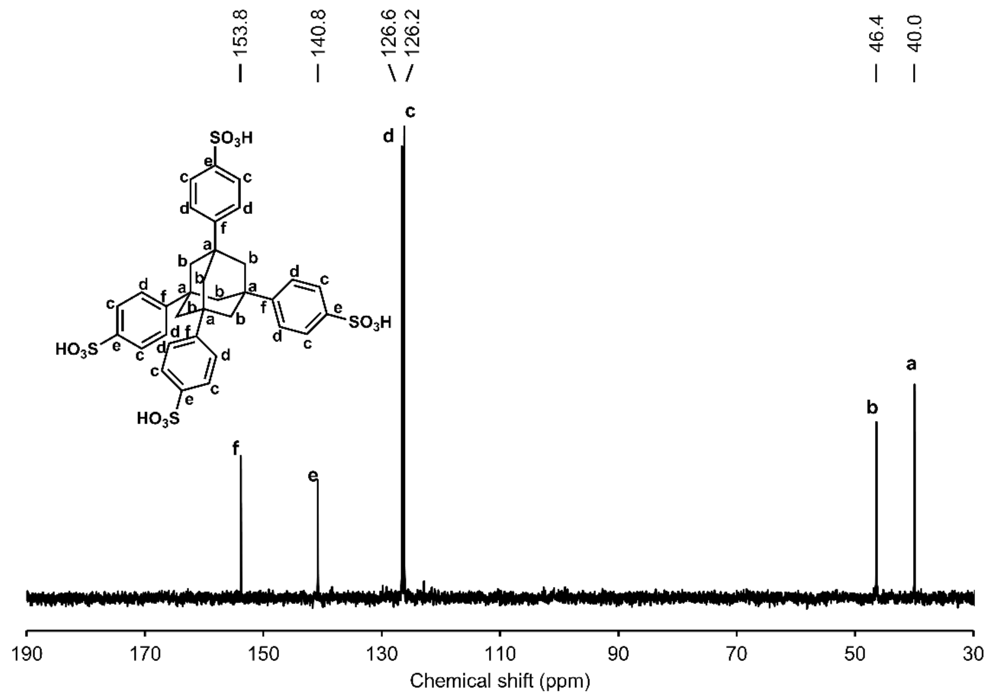

Figure S27. 400 MHz ^{13}C NMR spectrum (D_2O) of 4,4',4'',4'''-(adamantane-1,3,5,7-tetrayl)tetrabenzenesulfonic acid (**AdPS**): ^{13}C NMR (400 MHz, D_2O, δ): 153.8, 140.8, 126.6, 126.2, 46.4, 40.0.

Table S1. Structural properties of **AdPS/TPMA-F** reported by the Poreblazer software.[7]

Property	***dia***-topology	***lon***-topology	***uni***-topology
Minimum pore size (Å)	3.61	4.96	4.84
Maximum pore size (Å)	8.92	8.37	7.45

Table S2. Structural properties of **AdPS/TPMA-Cl** reported by the Poreblazer software.[7]

Property	***sod***-topology
Minimum pore size (Å)	5.88
Maximum pore size (Å)	15.7

Table S3. Structural properties of **AdPS/TPMA-Me** reported by the Poreblazer software.[7]

Property	***dia***-topology	***lon***-topology	***sod***-topology
Minimum pore size (Å)	3.66	5.65	4.57
Maximum pore size (Å)	6.25	7.06	15.6

Table S4. Surface area calculated by Brunauer–Emmett–Teller (**BET**) method and pore volume of **AdPS/TPMA-Me** based on the CO_2 isotherms.

	dia-topology	***lon***-topology	***sod***-topology
Surface area ($m^2\ g^{-1}$)	228	228	194
Pore volume ($cm^3\ g^{-1}$)	0.236	0.255	0.231

Table S5. Crystallographic Parameters of **AdPS/TPMA**.

	***dia*-topology (Two-fold)**
**formula**	$C_{27.5}H_{25}NO_3S$
**fw**	**449.54**
**crystal system**	**tetragonal**
**space group**	***I*-4*c*2**
***a* [Å]**	**18.6896(3)**
***b* [Å]**	**18.6896(3)**
***c* [Å]**	**24.9329(5)**
***α* [deg]**	**90**
***β* [deg]**	**90**
***γ* [deg]**	**90**
***V* [Å^3]**	**8709.1(3)**
Z	**16**
***T* [K]**	**213**
R_1 (I>2σ(I))	**0.0625**
**Rw (all data)**	**0.1644**
**CCDC no.**	**2238308**

Table S6. Crystallographic Parameters of **AdPS/TPMA-F**.

	dia-topology	*lon*-topology	*uni*-topology (left)	*uni*-topology (right)
formula	$C_{152}H_{88}F_{42}N_{10}O_{12}S_4$	$C_{36.7}H_{29.3}F_4N_{1.3}O_4S_{1.3}$	$C_{65}H_{58}F_6N_2O_7S_2$	$C_{65}H_{58}F_6N_2O_7S_2$
fw	3172.56	671.36	1157.25	1157.25
crystal system	monoclinic	hexagonal	trigonal	trigonal
space group	$P2_1$	$P6_3$	$P3_221$	$P3_121$
a [Å]	17.1130(2)	17.810(6)	27.3099(2)	27.3481(4)
b [Å]	25.1519(3)	17.810(6)	27.3099(2)	27.3481(4)
c [Å]	17.3707(2)	29.242(11)	17.1255(2)	17.1689(3)
α [deg]	90	90	90	90
β [deg]	93.3580(10)	90	90	90
γ [deg]	90	120	120	120
V [Å^3]	7463.94(15)	29552.6(15)	11061.5(2)	11120.6(4)
Z	2	6	6	6
T [K]	213	293	213	213
R_1 (I>2σ(I))	0.0701	0.1622	0.0422	0.0454
Rw (all data)	0.2108	0.4309	0.1335	0.1393
CCDC no.	2209700	2210216	2209701	2209702

Table S7. Crystallographic Parameters of **AdPS/TPMA-Me**.

	***dia*-topology**	***lon*-topology**	***sod*-topology**
**formula**	$C_{132}H_{138}N_4O_{12}S_4$	$C_{132}H_{140}N_4O_{12}S_4$	$C_{30.5}H_{31}NO_3S$
**fw**	**2100.70**	**2102.72**	**491.62**
**crystal system**	**monoclinic**	**monoclinic**	**cubic**
**space group**	$C2$	$P2_1$	$P4_232$
a **[Å]**	**26.390(17)**	**17.4923(5)**	**30.8219(6)**
b **[Å]**	**22.891(2)**	**28.7566(6)**	**30.8219(6)**
c **[Å]**	**25.764(16)**	**17.7263(4)**	**30.8219(6)**
α **[deg]**	**90**	**90**	**90**
β **[deg]**	**90.19(5)**	**118.527(3)**	**90**
γ **[deg]**	**90**	**90**	**90**
V **[Å^3]**	**15563.3(2)**	**7834.1(4)**	**29280.5(17)**
Z	**4**	**2**	**24**
T **[K]**	**213**	**213**	**213**
R_1 ($I>2\sigma(I)$)	**0.0695**	**0.0560**	**0.0932**
**Rw (all data)**	**0.2061**	**0.1562**	**0.2668**
**CCDC no.**	**2209746**	**2209699**	**2209745**

Table S8. Crystallographic Parameters of **AdPS/TPMA-Cl**.

	sod-topology
formula	$C_{27.5}H_{22}Cl_3NO_3S$
fw	552.87
crystal system	cubic
space group	$P4_232$
a [Å]	30.6107(3)
b [Å]	30.6107(3)
c [Å]	30.6107(3)
α [deg]	90
β [deg]	90
γ [deg]	90
V [$Å^3$]	28682.7(5)
Z	24
T [K]	213
R_1 (I>2σ(I))	0.1378
Rw (all data)	0.4033
CCDC no.	2190716

Table S9. Crystallographic Parameters of **AdPS/TPMA-Br**.

	sod-topology
formula	$C_{27.5}H_{22}Br_3NO_3S$
fw	686.25
crystal system	cubic
space group	$P4_232$
a [Å]	30.9171(9)
b [Å]	30.9171(9)
c [Å]	30.9171(9)
α [deg]	90
β [deg]	90
γ [deg]	90
V [Å^3]	29552.6(15)
Z	24
T [K]	213
R_1 (I>2σ(I))	0.1279
Rw (all data)	0.4063
CCDC no.	2190957

Table S10. Crystallographic Parameters of **AdPS/TPMA-I**.

	***sod*-topology**
**formula**	$C_{110}H_{88}I_{12}N_4O_{12}S_4$
**fw**	**3308.88**
**crystal system**	**trigonal**
**space group**	***R*3*c***
***a* [Å]**	**42.721(2)**
***b* [Å]**	**42.721(2)**
***c* [Å]**	**52.784(3)**
***α* [deg]**	**90**
***β* [deg]**	**90**
***γ* [deg]**	**120**
***V* [Å^3]**	**83430(8)**
Z	**18**
***T* [K]**	**173**
R_1 (I>2σ(I))	**0.1543**
**Rw (all data)**	**0.4080**
**CCDC no.**	**2191243**

References

1. V. A. Blatov, A. P. Shevchenko and D. M. Proserpio, *Cryst Growth Des*, 2014, **14**, 3576-3586.
2. S. Grimme, J. Antony, S. Ehrlich and H. Krieg, *J. Chem. Phys.*, 2010, **132**, 154104.
3. M. J. Frisch, G. W. Trucks, H. B. Schlegel, G. E. Scuseria, M. A. Robb, J. R. Cheeseman, G. Scalmani, V. Barone, G. A. Petersson, H. Nakatsuji, X. Li, M. Caricato, A. V. Marenich, J. Bloino, B. G. Janesko, R. Gomperts, B. Mennucci, H. P. Hratchian, J. V. Ortiz, A. F. Izmaylov, J. L. Sonnenberg, D. Williams-Young, F. Ding, F. Lipparini, F. Egidi, J. Goings, B. Peng, A. Petrone, T. Henderson, D. Ranasinghe, V. G. Zakrzewski, J. Gao, N. Rega, G. Zheng, W. Liang, M. Hada, M. Ehara, K. Toyota, R. Fukuda, J. Hasegawa, M. Ishida, T. Nakajima, Y. Honda, O. Kitao, H. Nakai, T. Vreven, K. Throssell, J. A. Montgomery, Jr., J. E. Peralta, F. Ogliaro, M. J. Bearpark, J. J. Heyd, E. N. Brothers, K. N. Kudin, V. N. Staroverov, T. A. Keith, R. Kobayashi, J. Normand, K. Raghavachari, A. P. Rendell, J. C. Burant, S. S. Iyengar, J. Tomasi, M. Cossi, J. M. Millam, M. Klene, C. Adamo, R. Cammi, J. W. Ochterski, R. L. Martin, K. Morokuma, O. Farkas, J. B. Foresman, and D. J. Fox, *Gaussian 16, Revision C.01*, Gaussian, Inc., Wallingford CT, 2016.
4. T. Ami, K. Oka, K. Tsuchiya and N. Tohnai, *Angew. Chem. Int. Ed.*, 2022, **61**, e202202597.
5. (a) J. Guo, Y. Wang, L. Feng, X. Zhong, C. Yang, S. Liu and Y. Cui, *Polym. Korea*, 2013, **37**, 437-441; (b) D. J. Hoffart, A. P. Cote and G. K. Shimizu, *Inorg. Chem.*, 2003, **42**, 8603-8605.
6. B. Lotz, *Chembiochem*, 2022, **23**, e202100658.
7. L. Sarkisov, R. Bueno-Perez, M. Sutharson and D. Fairen-Jimenez, *Chem. Mater.*, 2020, **32**, 9849-9867.
